# Positive maternal affect during mother–litter interaction is reduced in new mother rats exhibiting a depression-like phenotype

**DOI:** 10.1038/s41598-023-33035-z

**Published:** 2023-04-21

**Authors:** Idil Tuncali, Natalie Sorial, Kali Torr, Mariana Pereira

**Affiliations:** 1 Department of Psychological and Brain Sciences, University of Massachusetts Amherst, Amherst, MA 01003 USA; 2 Present Address: APDA Center for Advanced Parkinson Research and Precision Neurology Program, Harvard Medical School, Brigham and Women’s Hospital, Boston, MA 02115 USA

**Keywords:** Neuroscience, Social behaviour

## Abstract

The experience of positive affect during new motherhood is considered essential for a healthy mother–infant relationship, with life-long consequences for both mother and child. Affective availability and contingent responsiveness are often compromised in mothers experiencing postpartum depression, yet how maternal affect impacts parenting is not fully understood. In this study, we used the Wistar-Kyoto (WKY) rat model of depression and ultrasonic vocalizations to examine the relationship between maternal affect and parenting. We examined the affective and behavioral response of WKY and control new mother rats during social interactions with their offspring. Our results show that WKY mothers displayed altered USV signaling accompanying substantial disturbances in their maternal caregiving. In addition, WKY mothers failed to adjust vocal frequency in coordination with offspring proximity and interaction compared to control mothers. A follow up experiment demonstrated that the administration of the adenosine A_2A_ receptor antagonist MSX-3 ameliorated both maternal behavioral deficits and low positive affect in WKY mothers. Together, our results highlight the importance of maternal positive affect in the dyad relationship and suggest a role for the striatopallidal pathway in the affective processing of parenting.

## Introduction

The experience of positive affect during early new motherhood is considered fundamental for the healthy relationship between the mother and her infant, and the wellbeing of each^1–3^. Postpartum depression and other maternal neuropsychiatric disorders are characterized by low positive affect and attenuated feelings of pleasure and comfort with the infant, which has negative implications for the mother–infant relationship and the infant’s developmental outcome^4–10^. Mothers suffering postpartum depression are often less sensitive to their child’s needs and signals, vocalize less often, are affectively flat and less engaged during positive social interactions with their infants^11–15^. Despite the strong association between maternal affect and parenting, little is understood about how the affective experience of motherhood impacts parenting.

In humans, rats and other mammals, vocalizations are behavioral manifestation of affective states that critically organize social interactions^16,17^. Adult rats emit ultrasonic vocalizations (USVs) in a variety of contexts, classically subdivided into two major categories based on their average sound frequency^18–24^. Low-frequency “22-kHz” calls (range: 18–33 kHz with little or no frequency modulation) are typically emitted in aversive conditions, such as in anticipation of pain, during social defeat or drug withdrawal, and thus considered indicative of a negative affective state^25–34^. High frequency “50-kHz” USVs (range: 35–120 kHz with diverse spectrographic structure) are generally produced in appetitive situations, as during play, sexual interactions, or in anticipation of reward, and thus thought to reflect positive affect ^27,35–41^. Rat pups also emit USVs, mostly in the range of 30–65 kHz, when apart from their mother and/or littermates as well as during maternal handling, which critically coordinate maternal pup seeking and caregiving behaviors^42–54^. To date, only a couple of studies have examined the affective experience of new mother rats during social interactions with their offspring^55,56^.

In this study, we examined the relationship between maternal affect and parenting, including the impact of depressive-like symptomatology on mothers’ behavior and affective expression during interactions with their young. To this aim, we used the Wistar-Kyoto (WKY) rat strain, a well-validated animal model of depression that recapitulates core depressive-like neuroendocrine, neurochemical, and behavioral abnormalities, including severe disturbances in parenting, when compared to several control strains^57–63^. The first experiment examined the affective and behavioral response of WKY and control new mother rats during social interactions with their offspring. A follow-up experiment evaluated the ability of the adenosine A_2A_ receptor antagonist MSX-3 to ameliorate the behavioral deficits and low positive affect of WKY mothers.

## Results

### Experiment 1: Maternal affect during social interaction with offspring

To examine the affective experience of WKY and control Sprague–Dawley (SD) mother rats during interactions with their offspring, we recorded USVs from mothers and their offspring, separately and together in varying social contexts (Recordings (R) 1–6; see “Materials and methods”).

### Mother vocal repertoire

**Figure 1:**
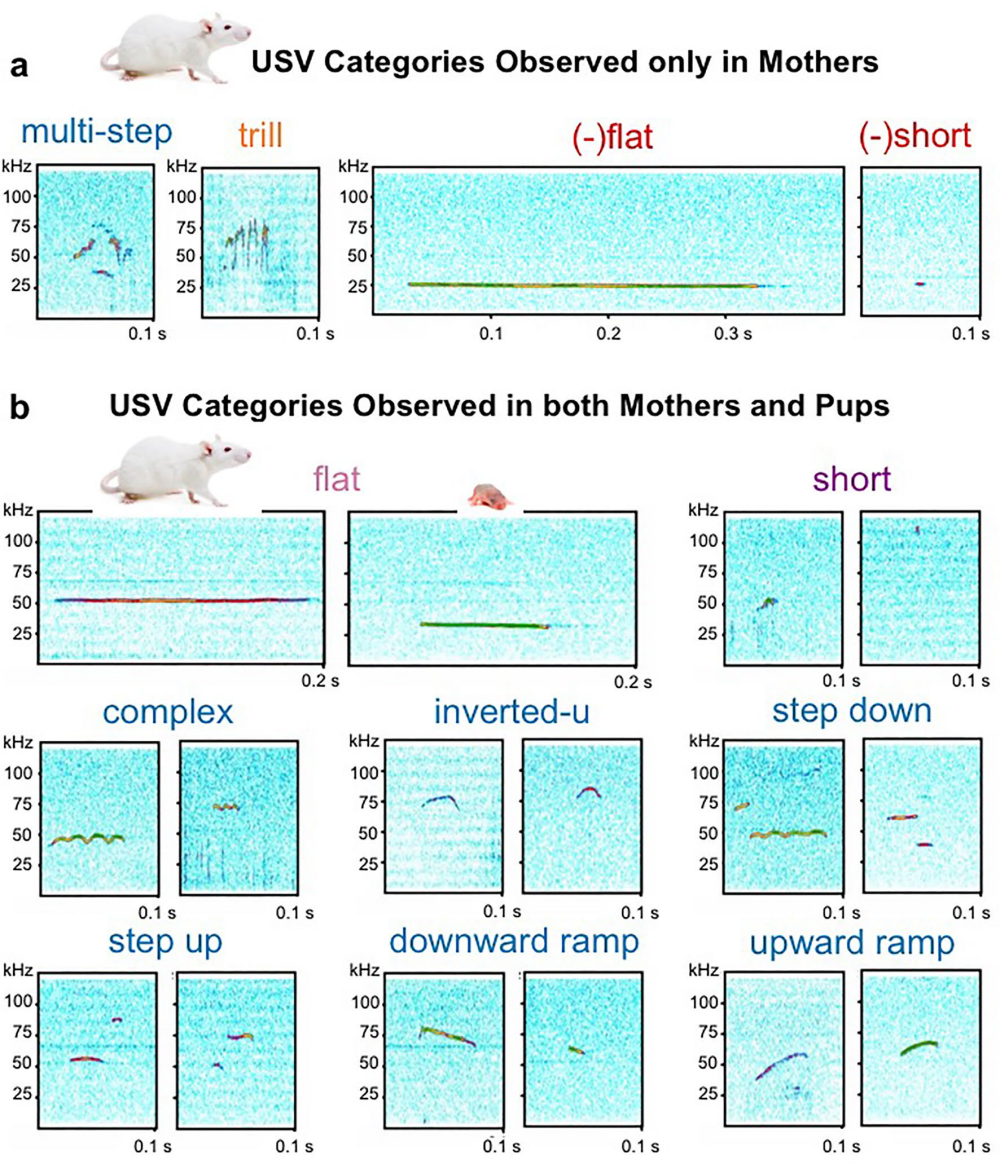
Representative spectrograms of USV call categories emitted by SD and WKY mothers and their PND7-8 pups (frequency is plotted along the y-axis and duration along the x-axis). (**a**) Multi-step and trill 50 kHz call categories and 22 kHz calls were only observed during mother-only recordings. (**b**) Flat, short and most FM calls were observed during both mother-only (R1 and R2) and offspring-only (R4, R5 and R6) recordings. *Flat*: A USV with near-constant frequency and a mean slope between − 0.2 and 0.2 kHz/ms. *Short*: A USV with a duration of less than 12 ms. *Complex:* A USV that contains two or more directional changes in frequency of at least 5 kHz each. *Inverted-u:* A USV with a steady increase followed by a steady decrease in frequency greater than 5 kHz each. *Step down:* A USV with an instantaneous frequency jump greater than 10 kHz to a lower frequency. *Step up*: A USV with an instantaneous frequency jump greater than 10 kHz to a higher frequency. *Downward ramp:* A USV with a steady decrease in frequency with a mean slope less than − 0.2 kHz/ms. *Upward ramp*: A USV with a steady increase in frequency with a mean slope greater than 0.2 kHz/ms. *Trill*: A USV with rapid oscillations in frequency. *Multi-step:* A USV with more than one instantaneous frequency jump greater than 10 kHz. *(–) Flat*: A USV below 30 kHz with near-constant frequency and a mean slope between -0.2 and 0.2 kHz/ms. *(–) Short:* A USV below 30 kHz and shorter than 12 ms.

**Figure 2:**
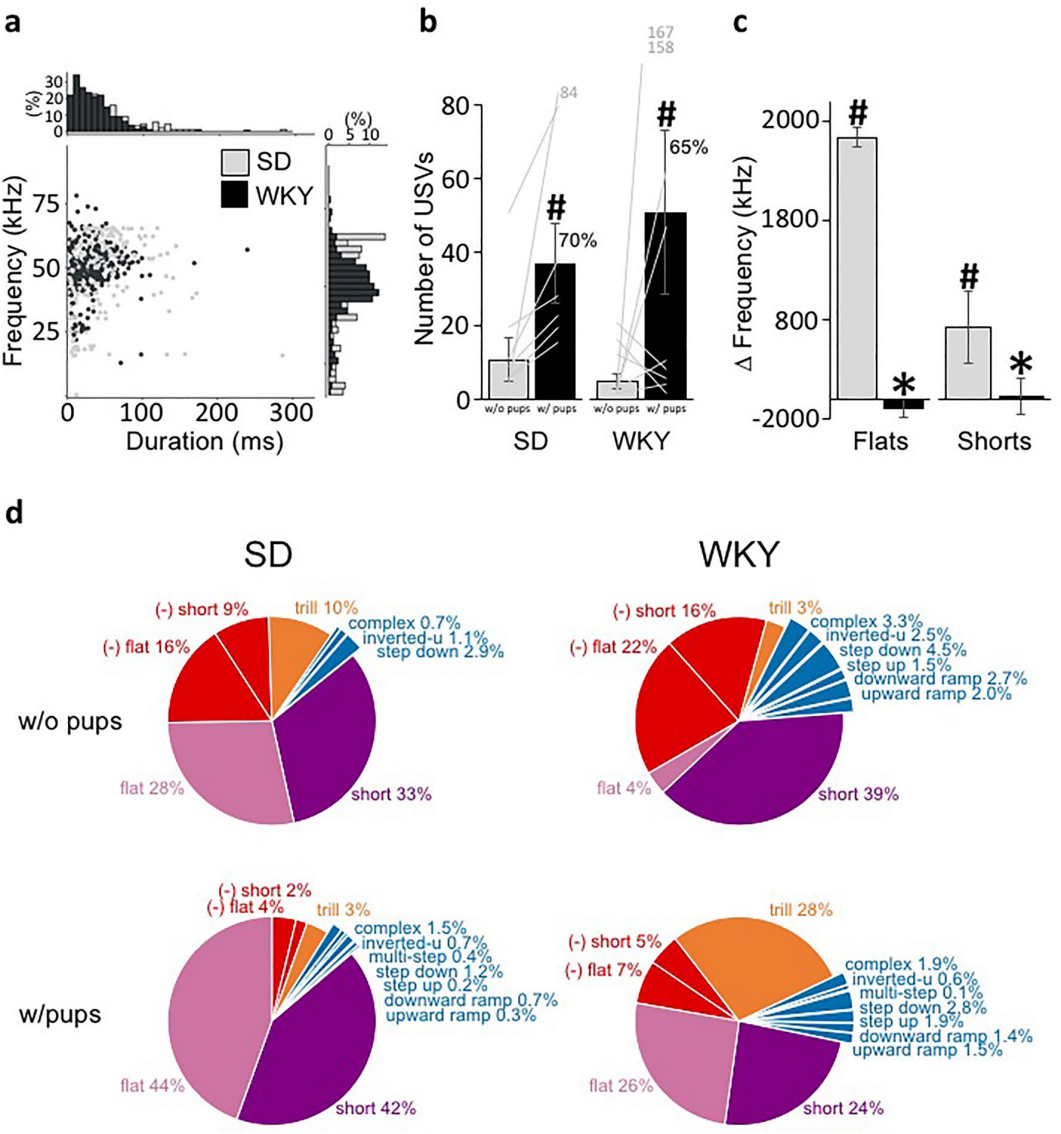
Maternal vocal repertoire. (**a**) Scatter plot of peak frequency vs. duration of USV calls emitted by SD and WKY mothers. Insert bar graphs denote the % frequency and duration distribution of calls. (**b**) Mean ± SEM number of USVs emitted by SD and WKY mothers when alone in the home cage without their offspring (R1) and with their offspring (R2) in the testing room (SD = 7 and WKY = 9). (**c**) Mean ± SEM change in frequency of flats and shorts from before to after the introduction of the offspring in the testing room. (**d**) Pie charts showing the proportion of USV call categories emitted by SD and WKY mothers when alone in the home cage (R1) and with their offspring in the testing room (R2). *denotes significant difference between strains. #denotes significant difference between recordings (within-strain comparison).

We first recorded the mothers alone in their cages for 5 min following a 10-min separation period from their offspring (R1: *Mother Alone*). Both SD and WKY mothers emitted USVs within 12 call categories previously described^64^, including negative 22 kHz calls, trills and other frequency-modulated (FM) 50 kHz calls, short and flat 50 kHz calls (Fig. 1). Figure 2a–d shows the number and acoustic characteristics of all call categories emitted by SD and WKY mothers. When alone in the room, SD and WKY mothers emitted similar categories and number of calls, although with a different call profile (SD vs WKY profile: Yates’ χ^2^ = 13.9, df = 4 p = 0.007; Fig. 2b). SD and WKY mothers showed a similar expression of negative and short calls, but SD mothers showed a higher percentage of flats, whereas WKY mothers showed a higher percentage of frequency-modulated (FM) calls (Fig. 2d).

#### SD and WKY mothers vocalize more when with their offspring

Immediately after R1: *Mother Alone*, the litter was returned to the testing room and placed next to the maternal cage, so that the mother was able to see, smell, and hear her pups, but not physically interact with them, and a second 5-min recording of the mother was taken (R2: *Mother with Litter Separated*). As shown in Fig. 2b, with the return of the offspring to the testing room, both SD and WKY mothers significantly increased the number of calls (Strain, F_(1,14)_ = 0.095 p = 0.76 ${\eta }_{p}^{2}$ = 0.007; Context, F_(1,14)_ = 6.3 p = 0.025 ${\eta }_{p}^{2}$ = 0.31; Strain × Context interaction, F_(1,14)_ = 0.5 p = 0.49 ${\eta }_{p}^{2}$ = 0.035), primarily due to an increased expression of 50 kHz calls (Strain, F_(1,14)_ = 0.18 p = 0.68 ${\eta }_{p}^{2}$ = 0.012; Context, F_(1,14)_ = 5.9 p = 0.029 ${\eta }_{p}^{2}$ = 0.29; Strain × Context interaction, F_(1,14)_ = 0.55 p = 0.47 ${\eta }_{p}^{2}$ = 0.038), although there was significant variability among WKY mothers. Specifically, only 4 of 9 WKY mothers vocalized more when with their offspring, whereas all 7 SD mothers did so (7/7 vs 4/9, Fisher’s Exact Test p = 0.034) (Fig. 2b). The expression of 22 kHz calls decreased, although it didn’t reach statistical significance (Strain, F_(1,14)_ = 0.15 p = 0.70 ${\eta }_{p}^{2}$ = 0.011; Context, F_(1,14)_ = 0.745 p = 0.4 ${\eta }_{p}^{2}$ = 0.05; Strain × Context interaction, F_(1,14)_ = 1.15 p = 0.30 ${\eta }_{p}^{2}$ = 0.076). Analysis of the number of calls in each category revealed a significant main effect of context in the number of flats (Strain, F_(1,14)_ = 0.23 p = 0.64 ${\eta }_{p}^{2}$ = 0.02; Context, F_(1,14)_ = 6.46 p = 0.023 ${\eta }_{p}^{2}$ = 0.32; Strain × Context interaction, *F*_(1,14)_ = 0.02 p = 0.89 ${\eta }_{p}^{2}$ = 0.002), and shorts (Strain, F_(1,14)_ = 0.23 p = 0.64 ${\eta }_{p}^{2}$ = 0.02; Context, F_(1,14)_ = 4.6 p = 0.05 ${\eta }_{p}^{2}$ = 0.25; Strain × Context interaction, *F*_(1,14)_ = 0.00 p = 0.99 ${\eta }_{p}^{2}$ < 0.001), with both SD and WKY mothers similarly emitting significantly more flats and shorts in the presence of their young.

The profile of call categories also changed for both SD (χ^2^ = 20.2, df = 4, p = 0.0005) and WKY (χ^2^ = 9.3, df = 4 p = 0.054) mothers (Fig. 2d). SD mothers reduced the expression of 22 kHz calls and produced proportionally more flats and shorts when with their pups (22 kHz, 25% to 6%; Flats, 28% to 44%; Shorts, 33% to 42%). WKY mothers also reduced the expression of negative calls, but in contrast to SDs, decreased the proportion of shorts and increased the emission of trills (22 kHz, 38% to 12%; Shorts, 39% to 24%; Trills, 3% to 28%). Thus, SD and WKY mothers expressed a different call profile in the presence of their offspring (χ^2^ = 14.5, df = 4, p = 0.006), with WKY mothers emitting a significantly lower proportion of flats (Strain, F_(1,14)_ = 10.5 p = 0.006 ${\eta }_{p}^{2}$ = 0.43; Context, F_(1,14)_ = 6.96 p = 0.019 ${\eta }_{p}^{2}$ = 0.332) and a significantly higher proportion of trills (Strain × Context interaction, *F*_(1,14)_ = 9.3 p = 0.009 ${\eta }_{p}^{2}$ = 0.4) compared to SD mothers. Furthermore, the majority of WKY mothers emitted trills (8 of 9), whereas only 1 of 8 SD mothers did (Fisher exact test statistic p = 0.0087).

#### SD mothers, but not WKY mothers, vocalize at a higher frequency when with their offspring

To examine whether mothers modify the acoustic properties of their USVs when in the presence of the offspring, we compared the peak frequency and duration of relevant call categories between recordings. SD mothers increased the peak frequency of their calls, including flats and shorts when with their offspring (Flats, F_(1,100)_ = 157.04 p = 0.000 ${\eta }_{p}^{2}$ = 0.61; Shorts, F_(1,18)_ = 5.8 p = 0.030 ${\eta }_{p}^{2}$ = 0.278), whereas WKY mothers did not (Flats, F_(1,72)_ = 0.03 p = 0.86 ${\eta }_{p}^{2}$ = 0.000; Shorts, F_(1,42)_ = 0.4 p = 0.53 ${\eta }_{p}^{2}$ = 0.009; Fig. 2c). Although, there was no difference in the duration of flats emitted by SD and WKY mothers between recordings, both SD and WKY mothers increased the duration of shorts when with their pups (Flats, SD: F_(1,100)_ = 0.014 p = 0.91 ${\eta }_{p}^{2}$ = 0.000; WKY: F_(1,72)_ = 0.005 p = 0.94 ${\eta }_{p}^{2}$ = 0.000. Shorts, SD: F_(1,18)_ = 6.6 p = 0.019 ${\eta }_{p}^{2}$ = 0.27; WKY: F_(1,42)_ = 7.7 p = 0.008 ${\eta }_{p}^{2}$ = 0.156).

### Offspring vocal repertoire

**Figure 3:**
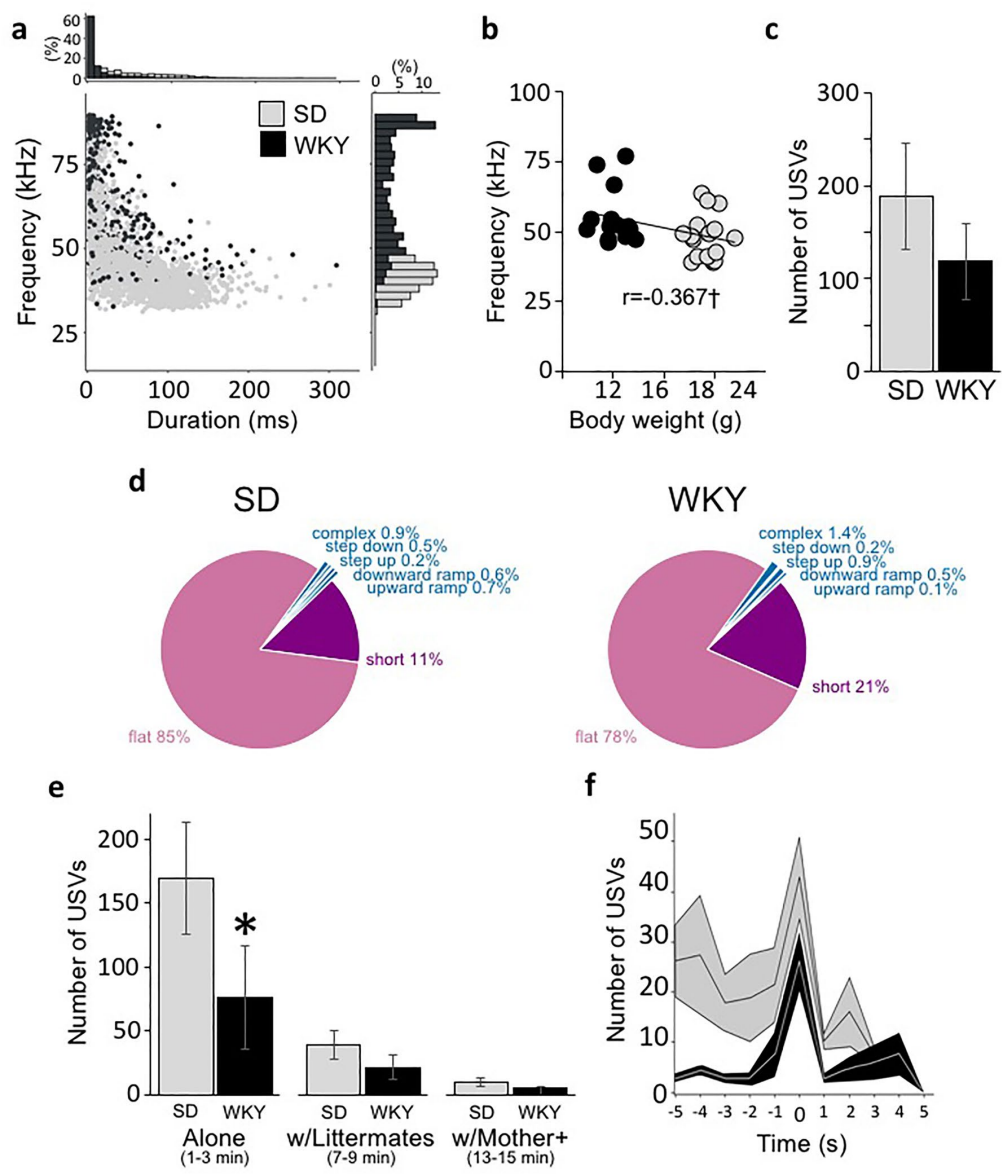

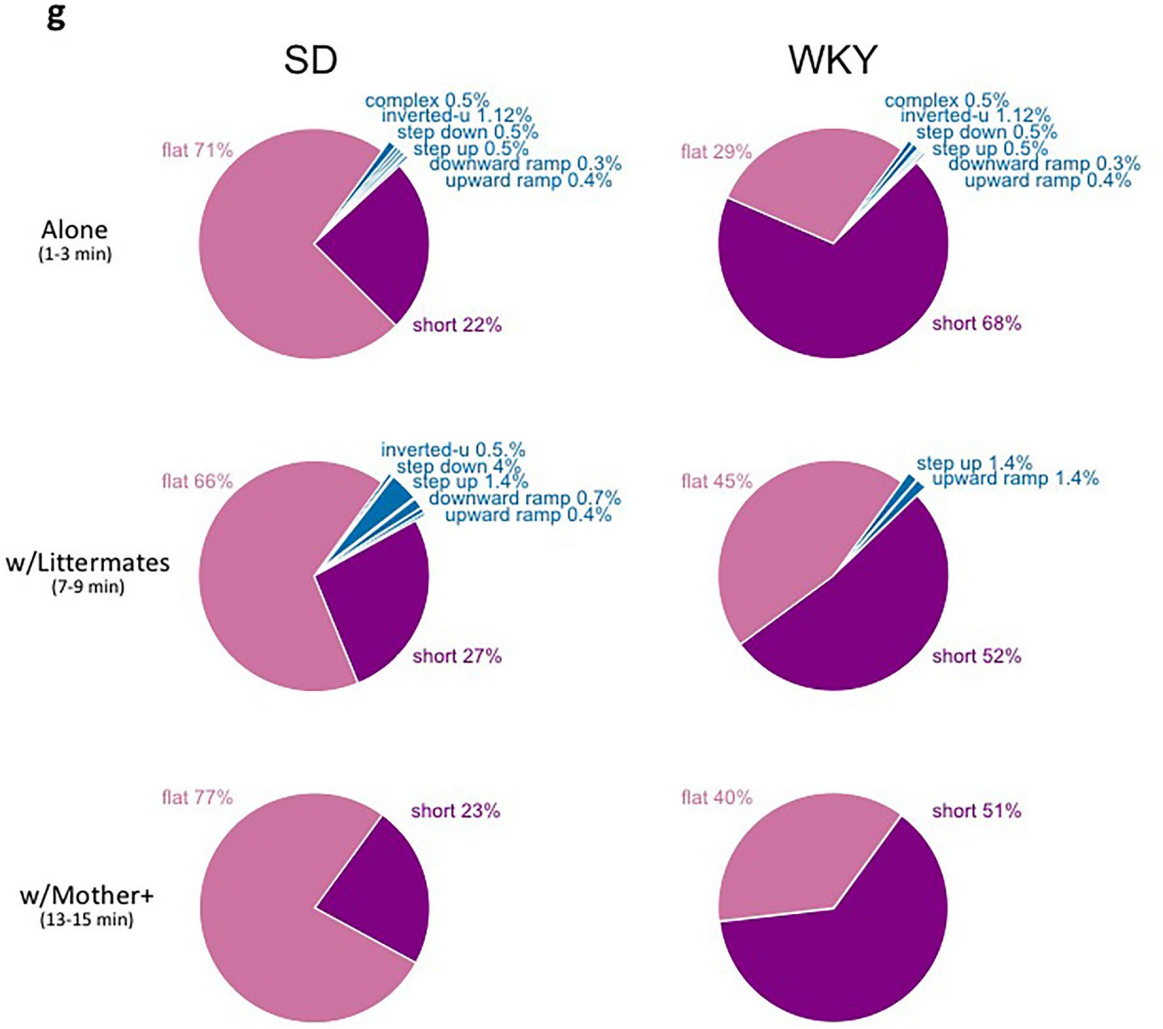
Offspring vocal repertoire. (**a**) Scatter plot of peak frequency and duration of USVs emitted by WKY and SD pups. Insert bar graphs denote the % frequency and duration distribution of calls. (**b**) Pearson correlation analysis of the peak frequency of calls with the body weight of SD and WKY pups. †denotes p < 0.05. (**c**) Mean ± SEM number of isolation-induced USV emitted by SD and WKY pups (R4/5) (SD_female_ = 8, SD_male_ = 8, WKY_female_ = 11 WKY_male_ = 11). No sex differences were detected, thus the data were pooled across sex within each strain for graphing purposes. (**d**) Pie charts showing the proportion of USV call categories emitted by isolated SD and WKY pups. (**e**) Mean ± SEM number of USVs emitted by SD and WKY litters in three social contexts, including when scattered apart, or grouped with a littermate, or with their mother and littermates in the nest (R6). (**f**) Mean ± SEM number of USVs triggered by manual retrieval (time: 0) in SD and WKY pups. (**g**) Pie charts showing the proportion of USV call categories emitted by SD and WKY pups in three social contexts, including when scattered apart, grouped with a littermate, and with their mother and littermates in the nest. *denotes significant difference between strains.

To define the vocal repertoire of offspring, we first recorded isolation-induced calls in randomly selected SD and WKY female and male pups (*R4 and R5: Male/Female Pup Isolated*). Immediately after the maternal behavior test, the mother and litter were removed from the home cage and placed in a new clean cage outside the testing room. For the isolation-induced recordings, one pup was placed back in the home cage outside of the nest quadrant and was recorded for 5 min. The pup was then returned to the mother, and a pup of the opposite sex was then placed alone in the home cage. After both a male and a female pup were recorded, the mother and pups were returned to the home cage. Figure 3a–d shows the number and acoustic characteristics of all call categories emitted by SD and WKY pups. Representative spectrograms for each of the call categories emitted by pups are displayed in Fig. 1, including FM, short and flat 50 kHz calls.

#### SD and WKY pups have a similar vocal reaction to isolation

In response to separation from their mother and littermates, both WKY and SD pups emitted a similar profile of USVs (χ^2^ = 5.5, df = 6, p = 0.48), mostly flat and short calls (Fig. 3d). The number of isolation-induced USVs (R4/5) was not different between WKY and SD male and female pups (Strain, F_(1,26)_ = 0.615 p = 0.44 ${\eta }_{p}^{2}$ = 0.024; Sex, F_(1,16)_ = 0.19 p = 0.67 ${\eta }_{p}^{2}$ = 0.008; Strain × Sex, F_(1,26)_ = 0.2 p = 0.66 ${\eta }_{p}^{2}$ = 0.008. SD_male_ 154.63 ± 89.26, SD_female_ 222.5 ± 76.04, WKY_male_ 128.57 ± 57.36, WKY_female_ 109.57 ± 58.78; Fig. 3c). Also, the latency to start calling (Strain, F_(1,26)_ = 1.18 p = 0.29 ${\eta }_{p}^{2}$ = 0.045; Sex, F_(1,16)_ = 0.4 p = 0.53 ${\eta }_{p}^{2}$ = 0.016; Strain × Sex, F_(1,26)_ = 1.3 p = 0.27 ${\eta }_{p}^{2}$ = 0.049), the total calling time (Strain, F_(1,26)_ = 0.26 p = 0.61 ${\eta }_{p}^{2}$ = 0.011; Sex, F_(1,16)_ = 0.86 p = 0.36 ${\eta }_{p}^{2}$ = 0.033; Strain × Sex, F_(1,26)_ = 0.13 p = 0.72 ${\eta }_{p}^{2}$ = 0.005), and the emission rate (number of calls/min: Strain, F_(1,26)_ = 1.3 p = 0.27 ${\eta }_{p}^{2}$ = 0.051; Sex, F_(1,16)_ = 0.43 p = 0.52 ${\eta }_{p}^{2}$ = 0.018; Strain × Sex, F_(1,26)_ = 0.26 p = 0.62 ${\eta }_{p}^{2}$ = 0.011) were not different between singly-isolated WKY and SD pups, again regardless of their sex.

#### WKY pups emit calls at a higher frequency

Analysis of the acoustic properties of the isolation-induced USVs revealed that WKY pups emitted isolation-induced calls at a higher peak frequency than SD pups (Flat Peak Frequency: Strain, F_(1,26)_ = 4.4 p = 0.045 ${\eta }_{p}^{2}$ = 0.15; Sex, F_(1,26)_ = 0.96 p = 0.34 ${\eta }_{p}^{2}$ = 0.04; Strain × Sex: F_(1,26)_ = 0.00, p = 0.98 ${\eta }_{p}^{2}$ = 0.000. Short Peak Frequency: Strain, F_(1,26)_ = 5.8 p = 0.024 ${\eta }_{p}^{2}$ = 0.18; Sex, F_(1,26)_ = 1.2 p = 0.68 ${\eta }_{p}^{2}$ = 0.007; Strain × Sex: F_(1,26)_ = 0.53, p = 0.48 ${\eta }_{p}^{2}$ = 0.02; Fig. 2a). However, flat and short call durations were similar between strains (Flat Duration: Strain, F_(1,26)_ = 0.68 p = 0.42 ${\eta }_{p}^{2}$ = 0.025; Sex, F_(1,26)_ = 1.9 p = 0.67 ${\eta }_{p}^{2}$ = 0.07; Strain × Sex: F_(1,26)_ = 0.008, p = 0.93 ${\eta }_{p}^{2}$ = 0.000. Short Duration: Strain, F_(1,26)_ = 0.81 p = 0.78 ${\eta }_{p}^{2}$ = 0.004; Sex, F_(1,26)_ = 0.04 p = 0.85 ${\eta }_{p}^{2}$ = 0.002; Strain × Sex: F_(1,26)_ = 0.18, p = 0.67 ${\eta }_{p}^{2}$ = 0.01; Fig. 3a).

Immediately after R4/5 isolation recordings, body weights and core temperatures were collected, as these variables are known to alter pup USV emission^46,48,65^. Pups were reunited with their mother immediately after examination. As expected, there was a significant difference in body weight between strains, but not between sexes, with WKY pups being significantly lighter than SD pups (SD_female_ 19.20 ± 0.29 g, SD_male_ 19.34 ± 0.45 g, WKY_female_ 11.99 ± 0.37 g, and WKY_male_ 12.08 ± 0.53 g. Strain, F_(1,26)_ = 304.9 p < 0.001 ${\eta }_{p}^{2}$ = 0.92; Sex, F_(1,26)_ = 0.071 p = 0.79 ${\eta }_{p}^{2}$ = 0.003; Strain × Sex interaction, *F*_(1,26)_ = 0.005 p = 0.94 ${\eta }_{p}^{2}$ < 0.0001). However, no strain or sex differences in body temperatures were detected (SD_female_ 32.80 ± 0.27 °C, SD_male_ pups 32.94 ± 0.32 °C, WKY_female_ 32.62 ± 0.34 °C, and WKY_male_ 32.76 ± 0.40 °C. Strain, F_(1,26)_ = 0.11 p = 0.74 ${\eta }_{p}^{2}$ = 0.004; Sex, F_(1,26)_ = 0.11 p = 0.75 ${\eta }_{p}^{2}$ = 0.004; Strain × Sex interaction, *F*_(1,26)_ = 0.28 p = 0.87 ${\eta }_{p}^{2}$ = 0.004]. Body weight was negatively correlated with the peak frequency of the USV emitted (Flats r = − 0.325, p = 0.079; Shorts r = − 0.417, p = 0.022; Fig. 3b), while no significant correlations between body weight or body temperature with the number or duration of these isolation-induced USVs were detected.

#### Social context impacts SD and WKY pups’ rate of USV emission

To further evaluate the offspring vocal repertoire, specifically whether offspring USVs change with social context, we recorded offspring’s USVs in the presence of their mother and littermates (R6: *Litter with Mother Anesthetized)*. Both mother and litter were removed from the maternal cage, the mother was anesthetized and immediately returned to the nest quadrant of the home cage. The litter was housed in a small cage until testing. Fifteen min later, an initial 5 min recording of the anesthetized mother verified the absence of maternal USVs. The pups were then scattered in the home cage away from the nest quadrant, and their USVs were recorded for 15 min.

As during R4/5 isolation recordings, SD and WKY pups emitted flat, short, and a variety of frequently modulated USVs, but no trills (Fig. 3g). To discern the effects of different social contexts on pup USV emission, the number, duration and frequency of the USVs were compared between minutes 1–3, 7–9, and 13–15 of the test. During the first 1–3 min, the pups were scattered throughout three quadrants of the cage without direct skin contact with their mother or littermates. By minutes 7–9, the pups had grouped together within their quadrant. Ten minutes into the recording, the litter was manually grouped around the anesthetized mother in the nest quadrant by the experimenter. Therefore, in minutes 13–15 of the recording, the pups were grouped with their anesthetized mother in the nest quadrant. The number of USVs similarly declined with increased social contact for both strains, but SD litters emitted significantly more USVs, especially flats, during the first 3 min of the recording compared to WKY litters (Flats: Strain, F_(1,13)_ = 12.65 p = 0.004 ${\eta }_{p}^{2}$ = 0.493; Context, F_(2,13)_ = 5.37 p = 0.035 ${\eta }_{p}^{2}$ = 0.292; Strain × Context, F_(2,13)_ = 5.03 p = 0.04 ${\eta }_{p}^{2}$ = 0.279. Shorts: Strain, F_(1,13)_ = 1.36 p = 0.264 ${\eta }_{p}^{2}$ = 0.095; Context, F_(2,13)_ = 7.52 p = 0.015 ${\eta }_{p}^{2}$ = 0.292; Strain × Context, F_(2,13)_ = 1.553 p = 0.235 ${\eta }_{p}^{2}$ = 0.107; Fig. 3e). Notably, once grouped with their mother and siblings, both SD and WKY pups emitted very few vocalizations (Fig. 3e).

#### Acoustic parameters of pup calls remain consistent across contexts and recordings

The duration of flat and short calls was similar between strains and did not change across social contexts (i.e., alone, with littermate, with littermates and mother. Flat Duration: Strain, F_(1,13)_ = 1.6 p = 0.23 ${\eta }_{p}^{2}$ = 0.11; Context, F_(2,13)_ = 2.8 p = 0.164 ${\eta }_{p}^{2}$ = 0.18; Strain × Context, F_(2,13)_ = 0.8 p = 0.45 ${\eta }_{p}^{2}$ = 0.058. Short Duration: Strain, F_(1,13)_ = 10.14 p = 0.008 ${\eta }_{p}^{2}$ = 0.482; Context, F_(2,13)_ = 0.89 p = 0.41 ${\eta }_{p}^{2}$ = 0.06; Strain × Context, F_(2,13)_ = 0.53 p = 0.48 ${\eta }_{p}^{2}$ = 0.04). Consistent with our previous results, WKY litters vocalized at higher frequencies than SD litters (Flat Frequency: Strain, F_(1,13)_ = 45.9 p < 0.001 ${\eta }_{p}^{2}$ = 0.852; Context, F_(2,13)_ = 1.29 p = 0.30 ${\eta }_{p}^{2}$ = 0.139; Strain × Context, F_(2,13)_ = 1.19 p = 0.31 ${\eta }_{p}^{2}$ = 0.130. Short Frequency: Strain, F_(1,13)_ = 8.2 p = 0.013 ${\eta }_{p}^{2}$ = 0.39; Context, F_(2,13)_ = 0.28 p = 0.67 ${\eta }_{p}^{2}$ = 0.021; Strain × Context, F_(2,13)_ = 0.13 p = 0.79 ${\eta }_{p}^{2}$ = 0.01).

In addition, the acoustic parameters of calls emitted by SD and WKY pups were similar between the two pup-only recordings (i.e., R4/5 vs. R6. Flat Duration: SD_R4/5_ 0.0604 ± 0.006 s, WKY_R4/5_ 0.061 ± 0.005 s, SD_R6_ 0.0568 ± 0.0036, and WKY_R6_ 0.0580 ± 0.0032 s. Strain, F_(1,13)_ = 0.32 p = 0.58 ${\eta }_{p}^{2}$ = 0.024; Recording, F_(1,13)_ = 0.001 p = 0.98 ${\eta }_{p}^{2}$ = 0.000; Strain × Recording, F_(1,13)_ = 0.020 p = 0.89 ${\eta }_{p}^{2}$ = 0.002. Short Duration: SD_R4/5_ 0.0063 ± 0.0004 s, WKY_R4/5_ 0.0063 ± 0.0006 s, SD_R6_ 0.00572 ± 0.00029 s, and WKY_R6_ 0.00551 ± 0.00032 s. Strain, F_(1,13)_ = 0.15 p = 0.71 ${\eta }_{p}^{2}$ = 0.01; Recording, F_(1,13)_ = 2.39 p = 0.15 ${\eta }_{p}^{2}$ = 0.155; Strain × Recording, F_(1,13)_ = 0.254 p = 0.623 ${\eta }_{p}^{2}$ = 0.019; Flat Frequency: SD_R4/5_ 42.96 ± 1.73 kHz, WKY_R4/5_ 49.07 ± 2.69 kHz, SD_R6_ 41.23 ± 1.28 kHz, and WKY_R6_ 48.89 ± 2.39 kHz. Strain, F_(1,13)_ = 6.7 p = 0.022 ${\eta }_{p}^{2}$ = 0.34; Recording, F_(1,13)_ = 0.001 p = 0.97 ${\eta }_{p}^{2}$ = 0.000; Strain × Recording, F_(1,13)_ = 0.489 p = 0.5 ${\eta }_{p}^{2}$ = 0.036. Short Frequency: SD_R4/5_ 52.55 ± 2.52 kHz, WKY_R4/5_ 58.64 ± 2.89 kHz, SD_R6_ 53.74 ± 4.56 kHz, and WKY_R6_ 59.25 ± 3.77 kHz. Strain, F_(1,13)_ = 8.53 p = 0.012 ${\eta }_{p}^{2}$ = 0.4; Recording, F_(1,13)_ = 1.76 p = 0.21 ${\eta }_{p}^{2}$ = 0.12; Strain × Recording, F_(1,13)_ = 0.538 p = 0.47 ${\eta }_{p}^{2}$ = 0.04).

#### Transport of pups to the nest enhances their USV emission

To mimic maternal retrievals, each pup was gently held by the neck and transported to the nest. Litters from both strains significantly increased their call rate during manual grouping compared to the emissions during the 60 s preceding it (Strain, F_(1,13)_ = 2.6 p = 0.13 ${\eta }_{p}^{2}$ = 0.17; Grouping, F_(1,13)_ = 14.94 p = 0.002 ${\eta }_{p}^{2}$ = 0.54; Strain × Grouping, F_(1,13)_ = 0.007 p = 0.93 ${\eta }_{p}^{2}$ = 0.001; Fig. 3f). Further analysis of USV parameters revealed no difference in the duration or peak frequency of the USVs emitted before and during manual grouping by WKY and SD litters (Flat Duration: Strain, F_(1,13)_ = 2.9 p = 0.11 ${\eta }_{p}^{2}$ = 0.18; Grouping, F_(1,13)_ = 0.081 p = 0.78 ${\eta }_{p}^{2}$ = 0.006; Strain × Time, F_(1,13)_ = 0.55 p = 0.47 ${\eta }_{p}^{2}$ = 0.04. Flat Frequency: Strain, F_(1,13)_ = 9.7 p = 0.008 ${\eta }_{p}^{2}$ = 0.43; Grouping, F_(1,13)_ = 1.3 p = 0.28 ${\eta }_{p}^{2}$ = 0.09; Strain × Grouping, F_(1,13)_ = 0.002 p = 0.96 ${\eta }_{p}^{2}$ = 0.000. Short Duration: Strain, F_(1,13)_ = 0.13 p = 0.73 ${\eta }_{p}^{2}$ = 0.01; Grouping, F_(1,13)_ = 0.001 p = 0.98 ${\eta }_{p}^{2}$ = 0.000; Strain × Grouping, F_(1,13)_ = 0.1 p = 0.79 ${\eta }_{p}^{2}$ = 0.006. Short Frequency: Strain, F_(1,13)_ = 2.8 p = 0.12 ${\eta }_{p}^{2}$ = 0.18; Grouping, F_(1,13)_ = 0.2 p = 0.66 ${\eta }_{p}^{2}$ = 0.02; Strain × Grouping, F_(1,13)_ = 0.6 p = 0.46 ${\eta }_{p}^{2}$ = 0.04).

### Maternal behavior and USVs during social interactions with offspring

To assess the affective state of SD and WKY mothers during maternal interactions with their offspring, we examined their USVs in a 30-min maternal behavior test (R3: *Mother–Litter Interaction)*.

#### WKY mothers exhibit deficits in their maternal behavior

**Figure 4:**
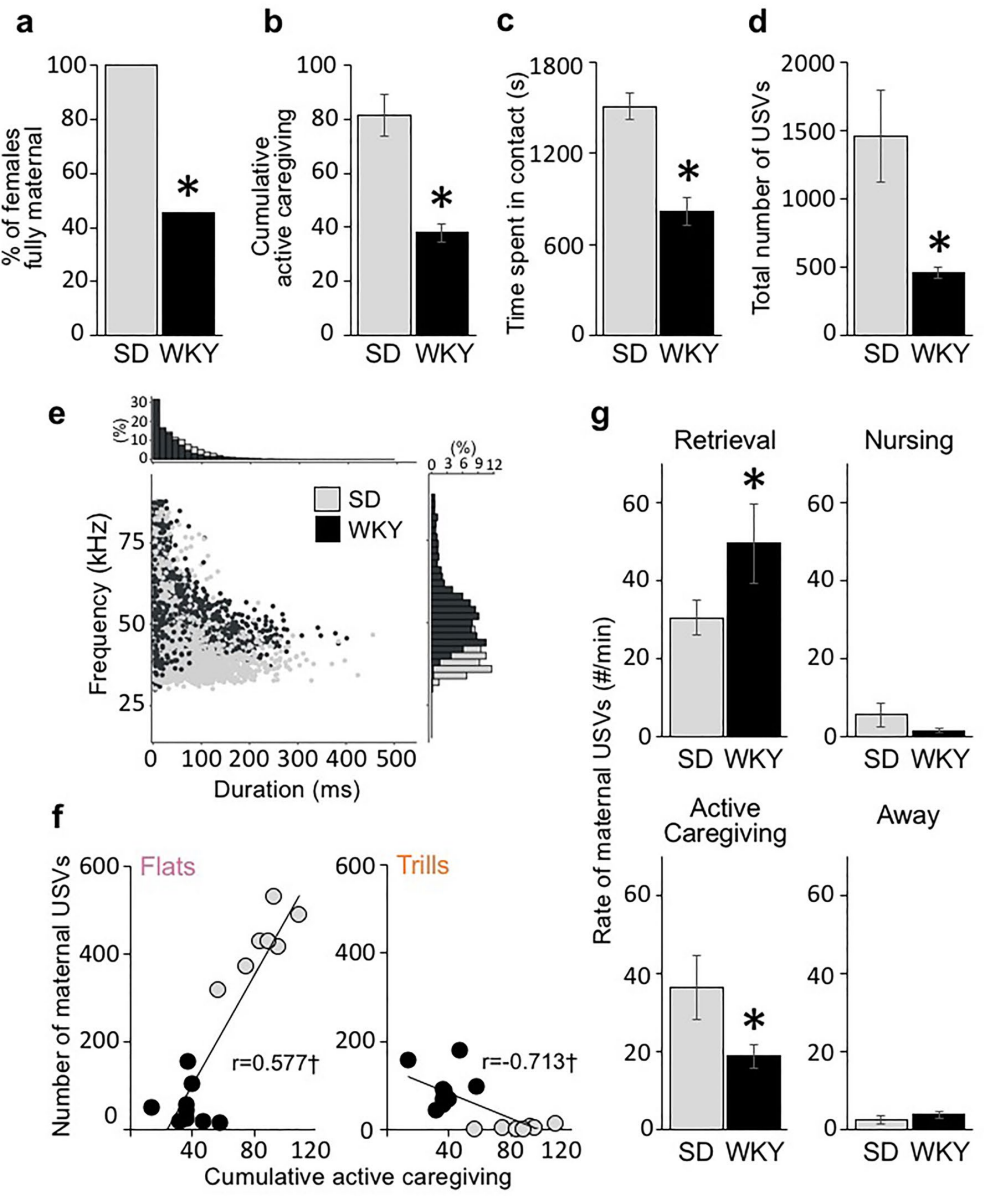

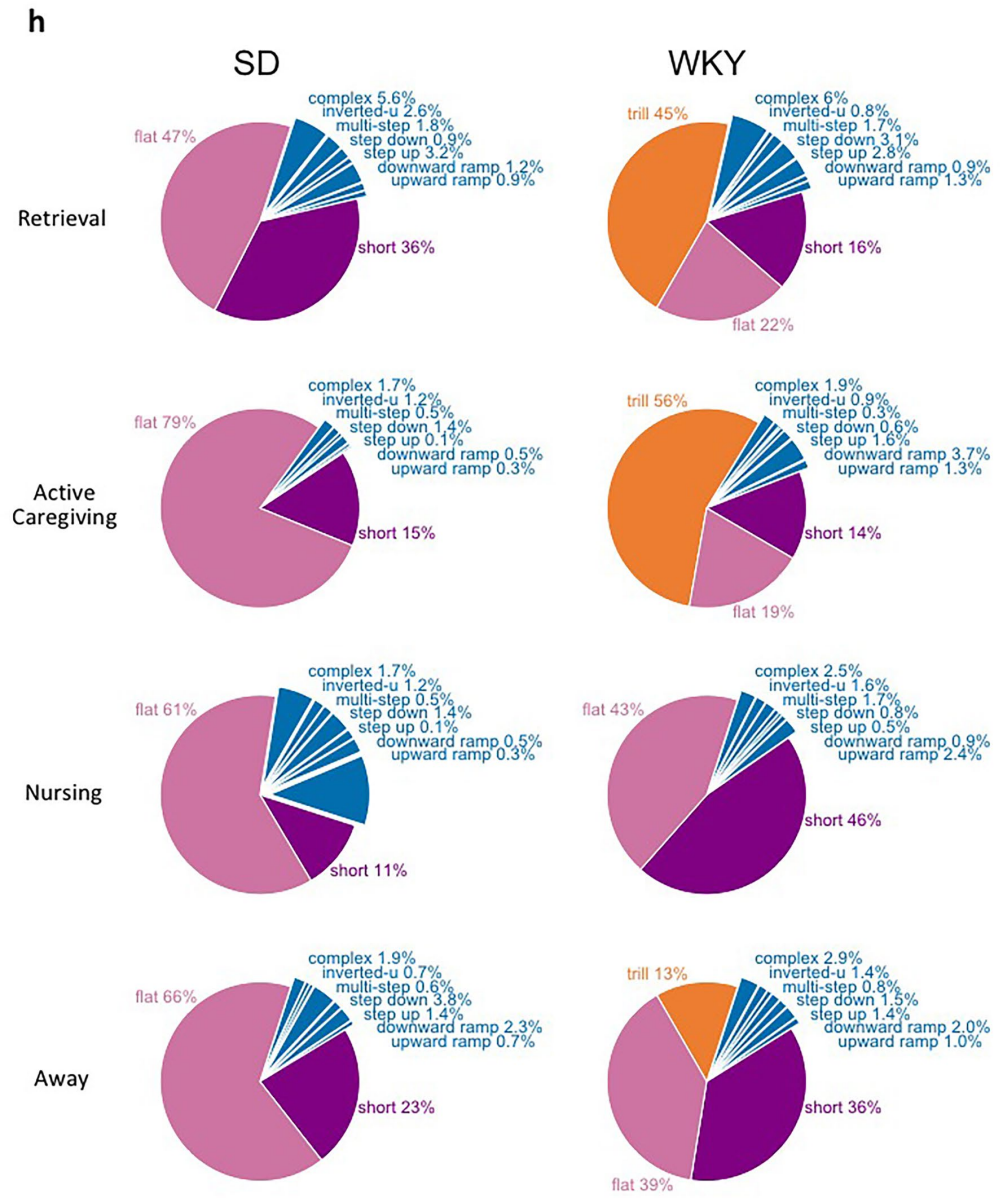
WKY mothers show reduced maternal responsiveness and affect during social interactions with their offspring. (**a**) Percentage of SD and WKY mothers retrieving and grouping all the pups and adopting a nursing posture during the 30-min maternal behavior test (SD = 8, WKY = 11). (**b**) Mean ± SEM cumulative active maternal behaviors of SD and WKY mothers during the 30-min maternal behavior test. (**c**) Mean ± SEM duration of time spent with pups by SD and WKY mothers during the 30-min maternal behavior test. (**d**) Mean ± SEM number of USV emitted by SD and WKY dyads during the 30-min recording (R3). (**e**) Scatter plot of peak frequency vs. duration of USV calls emitted by SD and WKY dyads. (**f**) Relationship between the number of maternal flats (left) and trills (right) and maternal behavior performance in SD and WKY mothers. Trendlines and Pearson’s r-values displayed along with scatter plot, ^†^denotes p < 0.05. (**g**) Mean ± SEM rate of maternal USVs (#/min) emitted during retrievals, active caregiving, nursing, or when away from the pups. (**h**) Pie charts showing the proportion of USV call categories emitted by SD and WKY mothers during retrievals, active caregiving, nursing, or when away from the pups during the maternal behavior test. *denotes significant difference between strains.

Consistent with previous results^63^, WKY mothers exhibited severe disturbances in their caregiving behavior compared to control SD mothers (Fig. 4a–c). SD mothers spent most of their time in contact with their young and exhibited robust expression of caregiving behaviors. In contrast, WKY mothers were abrupt and disorganized, often retrieving pups by body parts other than the typical nape of the neck (ratio of neck retrievals, SD 0.83 ± 0.05 and WKY 0.41 ± 0.14 , t(17) = 6.413 p = 0.000), spent significantly less time with their pups (SD 1508.4 ± 88.5 and WKY 817.8 ± 90.1, t(17) = 5.31 p = 0.000) and exhibited minimal active caregiving when with them (e.g., mouthing, SD 5.8 ± 1.1 and WKY 2.1 ± 1.1, t(17) = 2.23 p = 0.035; corporal licking, SD 34.3 ± 3.7 and WKY 12.6 ± 1.3, t(17) = 5.48 p = 0.000; anogenital licking, SD 23.5 ± 2.6 and WKY 7.1 ± 0.9, t(17) = 6.048 p = 0.000) (Fig. 4a–c). Only 5 of 11 WKY mothers retrieved and grouped all pups in the nest, licked their pups and nursed their litter during the test whereas all 8 SD mothers did so (5/11 vs 8/8, Fisher’s Exact Test p = 0.018) (Fig. 4a). In addition, WKY mothers exhibited fragmented patterns of caregiving behavior, spending a significant proportion of the test time away gathering scattered nesting material back to the nest (t(17) = − 2.805 p = 0.016) or engaging in non-maternal activities, such as resting (t(17) = − 3.92, p = 0.002) and feeding (t(17) = − 4.022 p = 0.003). Home-cage activity was not different between SD and WKY mothers (Crossing: SD 38.8 ± 3.3 and WKY 35.4 ± 2.5, t(17) = 0.86 p = 0.41; Rearing: SD 19.1 ± 2.9 and WKY 16.8 ± 1.8, t(17) = 0.97 p = 0.35; Self-grooming: SD 6.1 ± 0.9 and WKY 6.2 ± 0.8, t(17) = − 0.046 p = 0.96).

#### WKY dyads vocalize less during social interactions

All 12 50 kHz call categories occurred during the mother–young interaction recording, with both strains most prevalently emitting flat, short, trill, and other FM calls. No 22 kHz negative calls were detected during the mother–litter social interactions.

As shown in Fig. 4d,e, WKY dyads emitted significantly fewer USVs (t(17) = 3.48 p = 0.003), especially flats (t(17) = 3.7 p = 0.002) than SD dyads. In contrast, WKY dyads emitted more trills (t(17) = − 2.98 p = 0.008). All other call categories were similarly emitted by SD and WKY dyads (Short: t(17) = 0.84 p = 0.41; FMs: t(17) = 1.3 p = 0.21). The USV profile also differed between strains, with WKY dyads emitting a smaller proportion of flats and a higher proportion of shorts and trills than SD dyads (χ^2^ = 28.6, df = 6, p = 0.00007).

#### WKY mothers experience low positive affect during interactions with their offspring

Acoustic analysis of USVs in mother-only (R1 and R2) and offspring-only recordings (R4, R5 and R6) revealed differences between mothers and offspring in the mean duration and frequency of calls. Specifically, mothers emitted calls that were longer than those emitted by their offspring (e.g., Flat Duration: SD_MOTHER_ 0.0817 ± 0.0129 s vs SD_PUP_ 0.0568 ± 0.0036 s, t(12) = 1.844 p = 0.045; WKY_MOTHER_ 0.0771 ± 0.0094 vs WKY_PUP_ 0.0580 ± 0.0032 s, t(16) = 1.801 p = 0.045. Short Duration: SD_MOTHER_ 0.00812 ± 0.00109 s vs SD_PUP_ 0.00572 ± 0.00029 s, t(12) = 2.118 p = 0.028; WKY_MOM_ 0.00775 ± 0.00078 s vs WKY_PUP_ 0.00551 ± 0.00032 s, t(16) = 3.212 p = 0.003). In addition, mothers emitted flats at a higher frequency than their offspring (Flat Frequency: SD_MOTHER_ 52.79 ± 3.97 kHz vs SD_PUP_ 41.23 ± 1.29, t(12) = 2.780 p = 0.008; WKY_MOTHER_ 56.16 ± 2.99 vs WKY_PUP_ 48.89 ± 2.39, t(16) = 1.897 p = 0.038). Accordingly, upper bound cutoff durations (and frequency) of each USV call category emitted during the offspring-only recordings were used, within each dyad, to identify those USVs emitted by the mother during the 30-min social interaction with her offspring (e.g., within each dyad, flat calls in the R3 mother–litter recording with durations above R4-6 values were assigned to mothers). Similarly, upper bound cutoff frequencies were used to detect maternal flats. Lastly, trills and multi-steps were not observed during any offspring-only recordings, and thus calls within these USV categories were assigned to mothers (see Fig. 1). While we are aware our strategy likely didn’t account for all maternal calls, we are confident we identified the majority of the calls (> 85%) emitted by mothers (i.e., acoustic parameters, trills, minimal overlapping, etc. See below) during interactions with their offspring.

When only considering maternal USVs, a significant difference in the number of USVs emitted by SD and WKY mothers was found, with WKY mothers emitting significantly less calls than SD mothers during interactions with their offspring (t(17) = 2.878 p = 0.006; Fig. 4f,g). The USV profile also differed between strains, with WKY mothers emitting a smaller proportion of flats and a higher proportion of trills than SD mothers, particularly during retrievals and active caregiving (χ^2^ = 59.4, df = 3, p < 0.01 and χ^2^ = 90.5, df = 3, p < 0.01, respectively; Fig. 4h). In addition, a significant positive correlation was found between the maternal behavior performance and the number of flats (r = 0.577, p = 0.019). In contrast, a negative correlation was found between the maternal behavior performance and the number of maternal trills (r = − 0.713, p = 0.002) (Fig. 4f).

To determine the relationship between maternal USVs and caregiving behaviors, we analyzed the video recordings synchronized with the audio recordings. Maternal USVs during the following behavioral categories were examined: (i) retrievals (mother retrieves pups to the nest; (ii) active caregiving (mother is in the nest hovering over the pups, while performing active caregiving behaviors); (iii) nursing (mother adopts a quiescent nursing posture over the pups) and iv) away from pups (mother engages in non-maternal activities outside the nest). As expected, most calls during retrievals were emitted by pups. Most calls emitted thereafter were emitted by mothers during active caregiving, when all pups were grouped in the nest and emitted very few vocalizations (see R6 results above). This is further supported by the low proportion of overlapping calls once the litter was grouped in the nest (Behavior, F_(3,45)_ = 31.3 p < 0.001 ${\eta }_{p}^{2}$ = 0.65; retrieval vs active caregiving, SD: 31.39% vs 0.32% and WKY: 13.26% vs. 4.75%). There were significant effects of strain and behavior, and a significant strain × behavior interaction effect on the rate (#/min) of total, as well as flat and short maternal USVs (Total USVs: Strain, F_(1,15)_ = 21.1 p = 0.001 ${\eta }_{p}^{2}$ = 0.62; Behavior, F_(3,45)_ = 3.5 p = 0.08 ${\eta }_{p}^{2}$ = 0.22; Strain × Behavior, F_(3,45)_ = 56.85 p = 0.000 ${\eta }_{p}^{2}$ = 0.81. Flats: Strain, F_(1,15)_ = 21.1 p = 0.001 ${\eta }_{p}^{2}$ = 0.62; Behavior, F_(3,45)_ = 43.7 p = 0.000 ${\eta }_{p}^{2}$ = 0.77; Strain × Behavior, F_(3,45)_ = 19.9 p = 0.001 ${\eta }_{p}^{2}$ = 0.61). Shorts: Strain, F_(1,15)_ = 14.5 p = 0.002 ${\eta }_{p}^{2}$ = 0.53; Behavior, F_(3,45)_ = 28.8 p = 0.000 ${\eta }_{p}^{2}$ = 0.69; Strain × Behavior, F_(3,45)_ = 22.32 p = 0.000 ${\eta }_{p}^{2}$ = 0.63). SD mothers emitted a lower rate of USVs during retrievals but a higher rate of USVs during active caregiving than WKY mothers (p = 0.018 and p = 0.000, respectively) (Fig. 4g). SD mothers were responsible for most flats and shorts emitted during active caregiving, whereas less than 30% of calls belonged to SD mothers during retrievals (Flats, retrievals vs active caregiving p = 0.000; Shorts, retrievals vs active caregiving p = 0.000). In contrast, WKY mothers similarly emitted low rates of flats and shorts during interactions with pups (retrievals vs active caregiving both p = ns), which was significantly different compared to SD mothers (Flats, retrievals p = 0.048 and active caregiving p = 0.000; Shorts retrievals p = 0.94 and active caregiving p = 0.000). Of note, the higher rate of maternal USVs in WKY mothers during retrievals was mostly due to their higher emission of trills. SD and WKY mothers similarly emitted very few calls while away from their young or during nursing (p = 0.41 and p = 0.25, respectively; Fig. 4g).

In addition, SD mothers synchronized USV expression to moments of social interaction with their young, especially during active caregiving, whereas WKY mothers did not. Thus, SD mothers prevalently emitted most of their USV when with their young (vs away performing non-maternal active behaviors p < 0.001 and p = 0.001, respectively), whereas WKY mothers emitted similar USV rates across all behavioral categories (active caregiving vs away, p = 0.678; active caregiving vs nursing p = 0.480; nursing versus away, p = 0.12).

Of note, the number of USVs emitted, and the USV profiles, differed markedly when comparing the two mother–litter recordings (R3 vs R6). In the first 15 min of recording with an awake behaving mother (R3), dyads emitted approximately three times the average number of calls than dyads with an anesthetized mother (R6) (main effect of recording: F_(1,13)_ = 15.133, p = 0.002 ${\eta }_{p}^{2}$ = 0.538), indicating a significant maternal contribution to USVs. Moreover, the number of identified maternal USVs accounts for the difference between recordings.

### Experiment 2: Effect of the adenosine A_2A_ receptor antagonist MSX-3 on maternal behavior and USV emissions of WKY mothers

We then examined the ability of the adenosine A_2A_ receptor antagonist MSX-3 to ameliorate the behavioral deficits and low positive affect of WKY mothers. Separate groups of SD and WKY postpartum female rats were randomly assigned to receive IP injections of either saline vehicle (VEH) or 1.0 mg/kg of MSX-3 20 min prior to maternal behavior testing. A dose of 1.0 mg/kg of MSX-3 was chosen based on our published findings and preliminary data showing that it effectively ameliorated maternal behavior deficits in both SD mothers treated with D2 dopamine (DA) receptor antagonists and WKY mothers^66^.

#### MSX-3 ameliorates deficits in maternal behavior in WKY mothers

**Figure 5:**
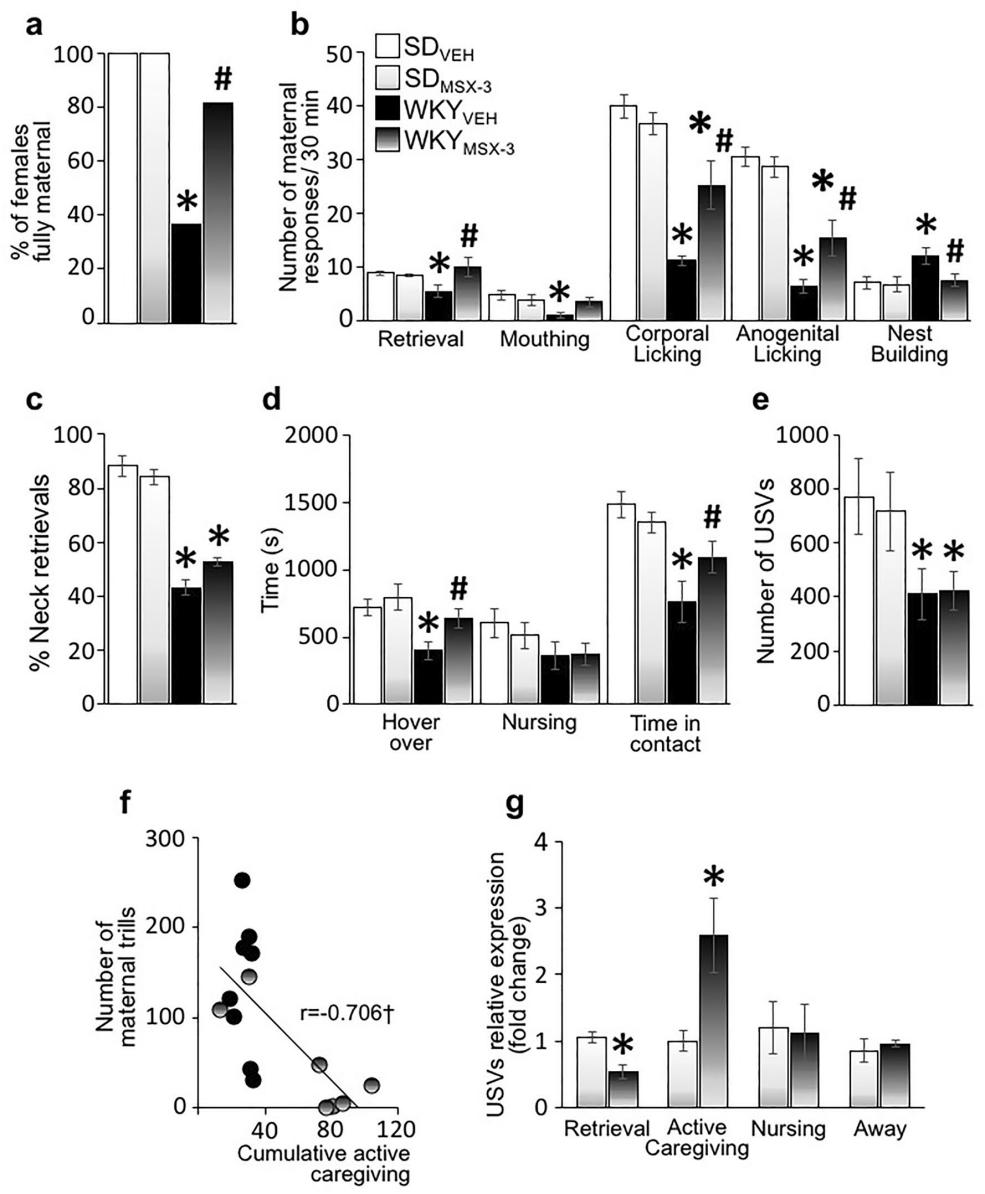

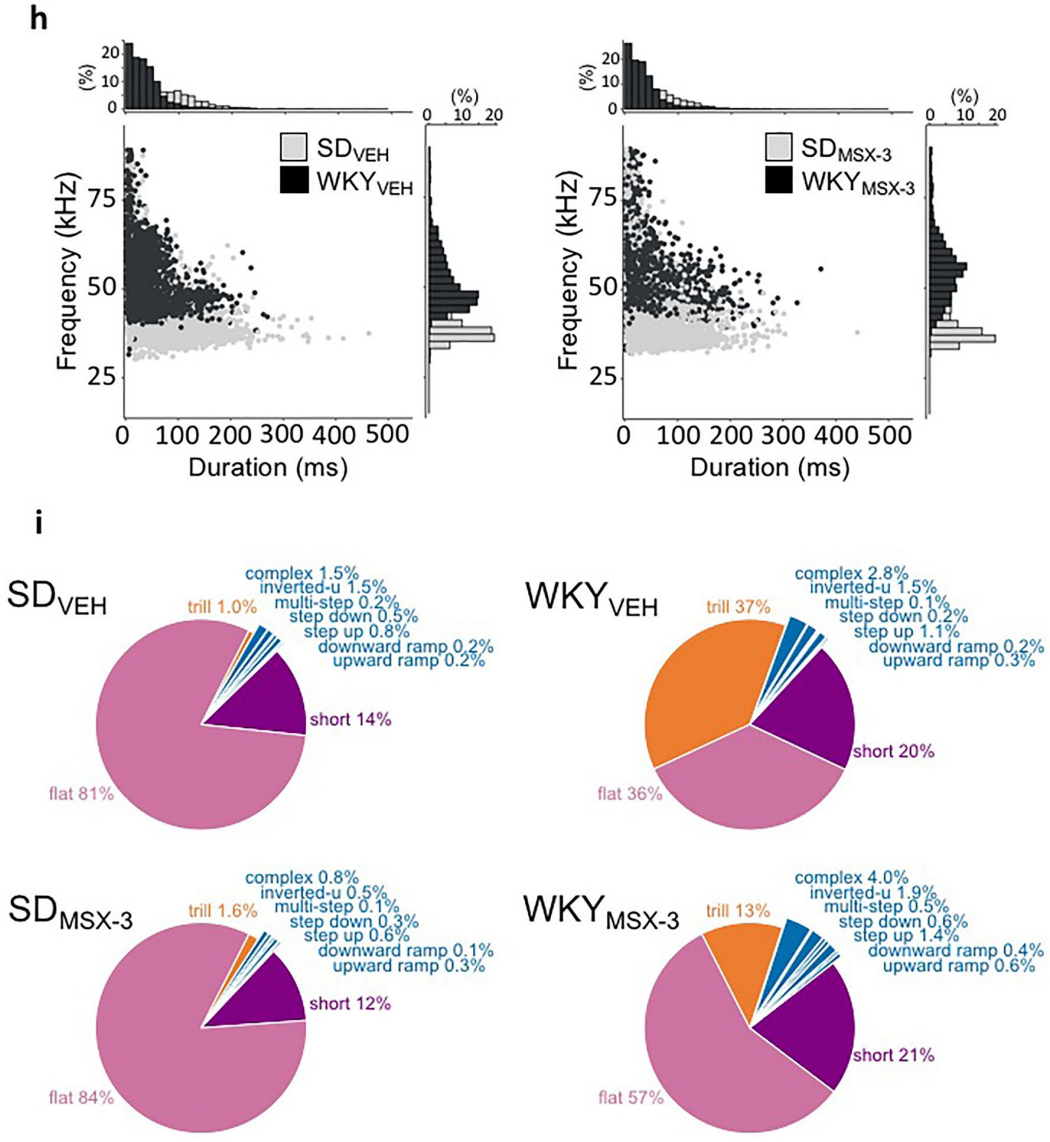
MSX-3 ameliorates deficits in maternal behavior and affect in WKY mothers. (**a**) Percentage of SD and WKY mothers retrieving and grouping all the pups and adopting a nursing posture during the 30-min maternal behavior test following treatment with MSX-3 or corresponding volume of vehicle (SD_VEH_ = 8, SD_MSX-3_ = 7, WKY_VEH_ = 9, WKY_MSX-3_ = 7). (**b**) Mean ± SEM number of active maternal responses over the 30-min maternal behavior test following treatment with MSX-3 or vehicle. (**c**) Ratio of neck retrievals over total number of retrievals. (**d**) Mean ± SEM duration of hover over, nursing and total time with pups over the 30-min maternal behavior test following treatment with MSX-3 or vehicle. (**e**) Mean ± SEM number of USV emitted by SD and WKY dyads during the 30-min recording following treatment with MSX-3 or vehicle. (**f**) Pearson correlation analysis of the number of maternal trills with maternal behavior performance of WKY mothers following treatment with MSX-3 or vehicle. †denotes p < 0.05. (**g**) Relative expression of USVs during retrievals, active caregiving, nursing, or when away from the pups. (**h**) Scatter plots of peak frequency vs. duration of USV calls emitted by SD and WKY dyads following treatment with MSX-3 (right) or vehicle (left). (**i**) Pie charts showing the proportion of call categories emitted during the 30-min maternal behavior test following treatment with MSX-3 or vehicle. *denotes significant between-strains difference in responding to control SD_VEH_ group. ^#^denotes significant within-strain difference in responding relative to vehicle group.

Consistent with our prior studies, the maternal behavior of vehicle-treated WKY mothers was minimal and disorganized compared to SD mothers (Fig. 5a–d). Administration of MSX-3 ameliorated deficits in several maternal caregiving components of WKY mothers (e.g., Corporal Licking, Strain, F_(1,38)_ = 102.2 p < 0.001 ${\eta }_{p}^{2}$ = 0.73; Treatment, F_(1,38)_ = 9.21 p = 0.004 ${\eta }_{p}^{2}$ = 0.195; Strain × Treatment, F_(1,38)_ = 21.64 p < 0.001 ${\eta }_{p}^{2}$ = 0.363; Latency to group, Strain, F_(1,38)_ = 25.58 p < 0.001 ${\eta }_{p}^{2}$ = 0.40; Treatment, F_(1,38)_ = 2.85 p = 0.1 ${\eta }_{p}^{2}$ = 0.07; Strain × Treatment, F_(1,38)_ = 9.147 p = 0.004 ${\eta }_{p}^{2}$ = 0.194; Total time in contact, Strain, F_(1,38)_ = 22.9 p < 0.001 ${\eta }_{p}^{2}$ = 0.38; Treatment, F_(1,38)_ = 4.83 p = 0.034 ${\eta }_{p}^{2}$ = 0.115; Strain × Treatment, F_(1,38)_ = 4.01 p = 0.05 ${\eta }_{p}^{2}$ = 0.98; Fig. 5a–d). MSX-3-treated WKY mothers more readily grouped all their pups into the nest (Latency to group p = 0.01), spent significantly more time with their pups (Time in contact p = 0.003), and licked and groomed them more (Corporal Licking: p < 0.001; Anogenital Licking: p < 0.001) than vehicle-treated WKY mothers, with expression levels comparable to those displayed by SD mothers (e.g., SD_VEH_ vs WKY_MSX-3_: Retrieval p = 0.89; Latency to group p = 0.161; Duration hover over p = 0.116). In addition, a significant majority of MSX-3-treated WKY mothers grouped all pups into the nest, licked and nursed their litters during the test compared to vehicle-treated WKY mothers (45% WKY_VEH_ vs 83% WKY_MSX-3_, 100% SD_VEH/MSX-3_ vs 83% WKY_MSX-3_, Fisher’s Exact Tests p = 0.09 and p = 0.481, respectively; Fig. 5a). In contrast, MSX-3 had no effect on the retrieval quality of WKY mothers, with most WKY mothers, regardless of treatment, retrieving pups by body parts other than the typical nape of the neck compared to SD mothers’ typical neck retrievals (% of neck retrievals: Strain, F_(1,38)_ = 227.8 p < 0.001 ${\eta }_{p}^{2}$ = 0.86; Treatment, F_(1,38)_ = 0.19 p = 0.66 ${\eta }_{p}^{2}$ = 0.005; Strain × Treatment, F_(1,38)_ = 1.3 p = 0.3 ${\eta }_{p}^{2}$ = 0.03; Fig. 5c).

MSX-3 treatment had no effect on the maternal behavior of SD mothers (SD_VEH_ vs SD_MSX-3_ all ps = ns; Fig. 5a–d). In addition, MSX-3 did not affect locomotor activity of SD and WKY mothers (Crossing, Strain, F_(1,38)_ = 1.3 p = 0.25 ${\eta }_{p}^{2}$ = 0.03; Treatment, F_(1,38)_ = 0.01 p = 0.92 ${\eta }_{p}^{2}$ = 0.000; Strain × Treatment, F_(1,38)_ = 0.11 p = 0.75 ${\eta }_{p}^{2}$ = 0.03; Rearing, Strain, F_(1,38)_ = 1.2 p = 0.27 ${\eta }_{p}^{2}$ = 0.03; Treatment, F_(1,38)_ = 0.66 p = 0.42 ${\eta }_{p}^{2}$ = 0.01; Strain × Treatment, F_(1,38)_ = 0.22 p = 0.6 ${\eta }_{p}^{2}$ = 0.06; Self-Grooming, Strain, F_(1,38)_ = 0.2 p = 0.65 ${\eta }_{p}^{2}$ = 0.005; Treatment, F_(1,38)_ = 0.17 p = 0.68 ${\eta }_{p}^{2}$ = 0.004; Strain × Treatment, F_(1,38)_ = 0.776 p = 0.38 ${\eta }_{p}^{2}$ = 0.02).

#### MSX-3 reduces the emission of trills in WKY mothers

Concomitant with changes in maternal behavior, administration of MSX-3 ameliorated the deficits in the expression rate and profile of USVs during social interactions with their young in WKY mothers (Fig. 5e–i). Consistent with results of experiment 1, vehicle-treated WKY dyads emitted significantly fewer flats and significantly more trills than SD dyads (SD_VEH_ vs WKY_VEH_: Flats p = 0.03, Trills p < 0.001; Fig. 5e). Administration of MSX-3 significantly reduced the emission of trills in WKY mothers (Trills: Strain, F_(1,38)_ = 21.25 p < 0.001 ${\eta }_{p}^{2}$ = 0.440; Treatment, F_(1,38)_ = 4.9 p = 0.035 ${\eta }_{p}^{2}$ = 0.16; Strain × Treatment, F_(1,38)_ = 6.23 p = 0.019 ${\eta }_{p}^{2}$ = 0.188, to levels similar to those of SD mothers (WKY_MSX-3_ vs WKY_VEH_ group p = 0.02; WKY_MSX-3_ vs SD_VEH/MSX-3_ both ps = ns; Fig. 5f–i). Collapsed across WKY groups, there was a significant negative correlation between the number of trills and maternal performance (r = − 0.706, p = 0.02), indicating once more the inverse relationship between maternal performance and trill emission (Fig. 5f). MSX-3 also significantly altered the call profile of WKY mothers, but not that of SD mothers, primarily by reducing the proportion of trills and increasing the proportion of flats (WKY_VEH_ vs WKY_MSX-3_ χ^2^ = 18.8, df = 4, p = 0.0008; SD_VEH_ vs SD_MSX-3_: χ^2^ = 1.7, df = 4, p = 0.79; Fig. 5i).

MSX-3 had no effect on any acoustic parameter of calls emitted by SD and WKY dyads (e.g., Flat Frequency: Strain, F_(1,27)_ = 0.3 p = 0.59 ${\eta }_{p}^{2}$ = 0.01; Treatment, F_(1,27)_ = 0.79 p = 0.38 ${\eta }_{p}^{2}$ = 0.03, Strain × Treatment, F_(1,27)_ = 0.4 p = 0.54 ${\eta }_{p}^{2}$ = 0.01. Flat Duration: Strain, F_(1,27)_ = 1.9 p = 0.18 ${\eta }_{p}^{2}$ = 0.06; Treatment, F_(1,27)_ = 0.04 p = 0.839 ${\eta }_{p}^{2}$ = 0.002; Strain × Treatment, F_(1,27)_ = 1.48 p = 0.23 ${\eta }_{p}^{2}$ = 0.052; Fig. 5h).

#### MSX-3 increases USV rate during social interaction in WKY mothers

Because MSX-3 did not affect the acoustic properties of the USVs emitted by SD and WKY dyads, strain-specific mean cutoff values determined in Experiment 1 were used to identify the USVs emitted by SD and WKY mothers in Experiment 2. Examination of USVs emitted by WKY mother during maternal caregiving categories revealed a significant effect of MSX-3 on the USV rate (Treatment, F_(1,14)_ = 4.41 p = 0.054 ${\eta }_{p}^{2}$ = 0.24; Behavior, F_(3,42)_ = 142.8 p < 0.001 ${\eta }_{p}^{2}$ = 0.9; Treatment × Behavior, F_(3,42)_ = 6.4 p = 0.23 ${\eta }_{p}^{2}$ = 0.31), with MSX-3 reducing the emission of trills during retrieval (p = 0.03) and increasing the emission of calls, mostly flats during active caregiving (p = 0.04) compared to the vehicle-treated WKY group (Fig. 5g,h). In contrast, MSX-3 had no effect on the USV expression of SD mothers in any behavioral category (Treatment, F_(1,13)_ = 0.2 p = 0.66 ${\eta }_{p}^{2}$ = 0.015; Behavior, F_(3,39)_ = 346.1 p < 0.001 ${\eta }_{p}^{2}$ = 0.96; Treatment × Behavior, F_(3,39)_ = 0.26 p = 0.65 ${\eta }_{p}^{2}$ = 0.02) (Fig. 5g,h).

Of note, compared to results from Experiment 1, Experiment 2 showed overall less USVs, regardless of strain and treatment, suggesting that the injection itself had an impact on USV expression (Fig. 5e). Notably, this reduction in USVs was mostly observed during active caregiving behavioral category, and not during retrievals, indicating that the mothers’ USVs, and not the offspring’s USVs, were affected by IP injection. This finding is consistent with previous reports that rats receiving IP injections call less^67^, and further supports the accuracy of our assignment of USVs to mothers. No differences were found between Experiments 1 and 2 for any maternal behaviors (i.e., SD vs SD_VEH_ and WKY vs WKY_VEH_, all ps = ns).

## Discussion

The present study shows that mother rats robustly emit 50 kHz USV calls during social interaction with their offspring, and that the number and profile of maternal USV calls predicts their maternal interest and caregiving efforts. Specifically, control SD mother rats emitted high rates of 50 kHz USVs, mostly flats and shorts, during contingent interactions with their offspring, which have been suggested to indicate positive affect. In addition, SD mothers adjusted the way they vocalize when with their offspring, by increasing the frequency and duration of their vocalizations. In contrast, WKY mothers exhibiting a depressive phenotype displayed substantial disturbances in maternal caregiving, fewer calls and an altered USV profile. Additionally, WKY mothers did not synchronize their USVs to moments of social interaction, nor did they change their vocal frequency when with their offspring, further indicative of disrupted interactions with their young. Administration of the adenosine A_2A_ receptor antagonist MSX-3 ameliorated both maternal behavioral deficits and low positive affect in WKY mothers. These results highlight the varying degree of positive affect that is experienced with new motherhood and the importance of maternal positive affect in the dyad relationship.

Consistent with prior work, our results find that WKY mothers exhibit severe deficits in their maternal behavior compared to control strains^57,63,68,69^. WKY females responded to being reunited with their pups following separation with minimal active caregiving, spending most of the test time away from their young. When interacting with their young, WKY mothers were less engaged and disorganized, and handled their pups more roughly, often dropping and/or stepping on pups, and retrieving pups by the head, limbs, side, or belly, instead of by the typical nape of the neck. This is in high contrast to SD mothers, who directed most of their behavior toward their pups, with little display of non‐maternal activities such as self‐grooming, resting and feeding during the test time. Our prior work has demonstrated that these caregiving differences between SD and WKY mothers are not driven by (i) differences in their locomotor activity, as both SD and WKY mothers are equally active in a 30-min test, (ii) by the handling associated with testing, or (iii) related to developmental or sensory characteristics of the pups, as they are also evident during undisrupted observations in the home cage and in cross-fostering experiments^63,70^. In this sense, clinical studies indicate that postpartum depression is significantly associated with less maternal responsiveness, and hostile and disengaged parenting^9,71,72^.

Both SD and WKY mothers exhibited a positive affective response during social interactions with their offspring, as indicated by 50 kHz USVs. Further supporting the positive experience of interaction was the emission of 22 kHz USVs, which are indicative of experiencing a negative emotional state^22^, uniquely during periods of separation from their young by both SD and WKY mothers. Consistent with published studies, following reunion with their offspring, both SD and WKY mothers emitted only 50 kHz calls and at a higher rate^55,56^, although there were substantial differences in their affective reaction. SD mother rats emitted high rates of 50 kHz calls, mostly flats and shorts, during contingent caregiving interactions with their young. In contrast, WKY mothers emitted significantly fewer calls and in a different profile, including fewer flats and more trills, and this altered USV profile was significantly correlated with their disrupted maternal performance. Significantly, trills were almost exclusively emitted by WKY mothers and mostly during exposure and interaction with scattered pups (i.e., during R2 and beginning of R3), indicating that WKY mothers exhibit a different emotional reaction to offspring’s calls than control SD mothers. In support, the number of trills emitted by WKY mothers predicted the severity of their parenting disturbances, with those WKY mothers emitting the highest trill rates, failing to group their pups in the nest and spending most of the test time away from them. Flat USVs are highly expressed during social interaction and have been suggested to be involved in social contact, coordination and reward^27,41^. Trill USVs, on the other hand, are commonly emitted during high emotional arousal in both appetitive and aversive situations^27,29,31,34,41,73–77^. Noteworthy, FM USVs that occur in aversive contexts are sensitive to anxiolytics and antidepressants^76–79^, further supporting their interpretation as signaling an anxiety-like state. Taken together, our results suggest that WKY mothers might find the demands of caregiving overwhelming and experience low positive affect during social interactions with their offspring, which strongly correlates with their rough and fragmented maternal behaviors. In support, baby cries produce amplified feelings of anxiety and panic in mothers with depression, who are more likely to perceive their child as difficult and to respond with irritability when their children make normal demands of care^9,80–83^.

Significantly, SD mothers, but not WKYs, shifted to a higher vocal frequency when with their offspring. In addition to this vocal shift, SD mothers synchronize their USVs to moments of social interactions with their offspring, further suggesting that these maternal calls are indicative of the mother’s positive affect. In contrast, WKY mothers lacked coordination of vocalizations with social interacting moments. This result is consistent with clinical studies showing that mothers experiencing postpartum depression speak less to their infants and are less likely to utilize offspring-directed vocalization (‘motherese’ or ‘baby talk’) during interactions with their infants^84,85^. In humans and other mammals, the use of ‘motherese’ is thought to indicate affect and to promote social interactions that are critical for the affective, cognitive, and social development of the offspring^84,86–90^.

The reduced caregiving and positive affect of WKY mothers was accompanied by their offspring’s altered USVs, potentially reflecting disrupted affiliative behavior of WKY pups related to the ongoing insensitive interaction style of their mothers. Although SD and WKY pups emitted similar call rates and profiles throughout most of the social conditions examined, WKY pups showed altered calling patterns when scattered in the home cage away from the nest and their mother. In agreement, one previous report showed reduced maternal potentiation of USVs and proximity-seeking behaviors in WKY pups^91^. Likewise, several reports have shown that rat pups alter their vocalizations when exposed to infrequent and/or rough maternal interactions^43,52,92^. Notably, clinical studies have shown a similar pattern, with infants of mothers suffering from depression being more likely to display an insecure attachment to their mothers than infants of control mothers^93–95^. It is also likely that offspring behavior in turn influences the mother’s maternal responsiveness and affective state, promoting a cycle of dysfunctional interactions. Unfortunately, limited studies have examined infant influences on their mother's affective well-being^96^. Future studies should consider the dynamic and reciprocal nature of the mother–infant relationship to better understand the bidirectional influences on the dyad’s affective wellbeing.

In addition, WKY pups’ higher peak frequency of calls were consistent across all recordings, suggesting inherent differences between strains. Consistent with our prior study, WKY pups gain weight, reach developmental milestones, and thermoregulate similarly to SD pups^63^, arguing against a developmental delay. One likely explanation, however, is related to their size difference, as WKY pups are ~ 7 g smaller than SD pups. In support, and consistent with a previous study^97^, a significant negative correlation was found between body weight and the frequency of the USVs emitted by rat pups. In addition, acoustic parameters of USVs, including peak frequency, change as the pups grow and develop^43,45,98–100^.

Otherwise, SD and WKY pups had similar call rates and profiles throughout the different social contexts examined. These results are highly consistent with previous work highlighting isolation-induced vocalizations in rat pups and the contact quieting response following reunion with littermates and mother, as well as the increase in USVs rates during retrieval, regardless of whether retrievals are performed by the mother or by an experimenter^43,52,101^. Our finding that both SD and WKY pups similarly emitted little calling while in the nest also confirms prior reports^102,103^.

Administration of the selective A_2A_ receptor antagonist MSX-3 substantially ameliorated the active caregiving deficits of WKY mothers, to levels characteristic of SD mothers. Thus, MSX-3-treated WKY mothers readily approached their pups and spent most of the test time with their young, actively taking care of them. This result is consistent with previous findings demonstrating a reversal effect of MSX-3 in motivational deficits induced by DA antagonism, including haloperidol-induced deficits in maternal behavior^66,104–106^. Notably, WKY’s caregiving deficits resemble those of SD mothers following systemic and intra-accumbens administration of a DA receptor antagonist^66,107–111^, indicative of the magnitude of the caregiving deficits in WKY mothers. Adenosine A_2A_ receptors are almost exclusively expressed in the striatum and highly colocalized with DA D2 receptors on GABAergic striatopallidal neurons, where they antagonize DA D2 receptor activity^112–117^. Consistent with prior work, administration of MSX-3 reversed the effects of haloperidol, but was without effect when administered alone, on the maternal behavior of SD mothers, suggesting that A_2A_ antagonists are effective in conditions of reduced DAergic activity but not under ‘normal’ conditions^66,118,119^. Taken together, these results suggest that blunted mesolimbic DAergic responsiveness to offspring underlies aspects of the deficits in active caregiving of WKY mothers. In agreement, WKY mothers have lower intracellular levels of all monoamines, including dopamine, as well as different patterns of change in their monoamine pathways as they transition across postpartum, compared to SD mothers^63^.

MSX-3 treatment also reduced the emission of trills and synchronized maternal USVs to moments of interaction with the offspring (i.e., increased the rate of calls, mostly of flats and shorts, during active caregiving) in WKY mothers, indicative of improved positive affect. Moreover, the reduced expression of trills correlated with improved maternal performance in WKY mothers, suggesting that the low positive affect and reduced motivational aspects of caregiving in WKY mothers are related to increased striatopallidal activity. This result is consistent with prior work demonstrating that activation of A_2A_ receptors with CGS21680 disrupts behavioral activation functions of motivated behavior and attenuates the emission of pro-social 50 kHz USVs in rats^120^. Similarly, the production of USVs in appetitive contexts is strongly related to the activity of VTA → NA DA neurons^121^. Of note, MSX-3 did not impact the USV expression or behavior of SD mothers, consistent with previous findings^119,120^. Taken together, reduced striatopallidal activity with MSX-3 ameliorated both maternal affect and caregiving in WKY mother, suggesting overlapping neurobiology. In support, effort-related motivational symptoms, well-recognized, highly debilitating aspects of depression, are highly correlated to problems with low positive affect and social function, and treatment strategies that target these symptoms have shown to be beneficial to positive affect and emotional wellbeing^118,122–126^. Moreover, positive affect has been associated with increased working memory, cognitive flexibility and effort-related functions^127–129^, all processes key to parenting, suggesting a bidirectional benefit between maternal affect and parenting.

In conclusion, this study recapitulates the observed variations in positive affect experienced with new motherhood in humans, and suggests a common neurobiological substrate underlying maternal affect and contingent responsiveness toward offspring. Understanding how the maternal brain manifests positive affect and the neurobiological mechanisms by which maternal affect impacts parenting is not only essential to our understanding of how mothers mother, but also for developing more effective intervention strategies for depression and other postpartum neuropsychiatric disorders aimed at restoring the mother–infant relationship.

## Materials and methods

### Animals

Primiparous postpartum Sprague–Dawley (SD) and Wistar–Kyoto (WKY) female rats purchased from Charles River Laboratories (Kingston, NY) and approximately 90 days of age were used. Both SD and WKY strains are derived from the Wistar strain, and thus SDs have consistently been used as a control strain for the WKY strain^58,60–63^. Animals were maintained on a 12/12 h light/dark cycle (lights on from 7:00 A.M. to 7:00 P.M.) at 22 ± 1 °C with ad libitum access to food, water and sunflower seeds. Experienced SD and WKY male rats, ranging from 90 to 180 days of age, were used for mating in our laboratory. Before giving birth, pregnant females were housed in individual clear Plexiglass cages (38.5 cm × 48.5 cm × 20.5 cm) lined with fresh Sani-Chips^®^ bedding and containing Eco-Bedding nest-building material. On postpartum day (PPD) 1 (birth = day 0) litters were culled to 8 pups (3–5 males, 3–5 females) per mother rat. All experimental procedures followed the ARRIVE guidelines, were performed in compliance with the guidelines of the NIH Guide for the Care and Use of Laboratory Animals, and with the approval of the Institutional Animal Care and Use Committee at the University of Massachusetts Amherst.

### General procedure

All behavioral procedures were conducted during the light phase of the light/dark cycle. One day before testing, a 5-cm high Plexiglas divider was inserted into each female’s cage to divide the floor of the cage into four equal compartments. Dyads were tested in their home cage, which was placed into an adjoining testing room 15 min prior to starting the test. Room temperature was maintained at 22 ± 1 °C. A microphone was positioned above the cage, and a video camera recorded the behavior of the mother and/or pups for later offline analysis. Once the day’s recordings were completed, females and their pups were returned to the colony room.

### Experiment 1: Maternal affect during social interaction with offspring

This experiment examined the affective responses of SD and WKY mother rats during social interaction with their offspring. In fast-paced, dynamic social interactions, it can be difficult to readily attribute calls to a specific member of the mother–infant dyad. In order to determine maternal vocalizations, PPD7-8 WKY and SD mothers and their pups underwent a series of recordings.

Day 1 of testing began with the litter removed from the home cage and housed in a small cage (lined with bedding and containing nest-building material from the maternal cage) outside of the testing room. Ten minutes after the removal of the litter, a 5-min recording of the mother alone in her cage was taken (R1: *Mother Alone).* The cage containing the litter was then returned to the testing room and placed adjacent to the maternal home cage, so that the mother was able to see, smell, and hear her pups, but not physically interact with them, and a second 5-min recording of the mother was taken (R2: *Mother with Litter Separated).* Thereafter, the litter was scattered in the home cage opposite to the nest, and a 30-min recording was taken in conjunction with a maternal behavior test (R3: *Mother–Litter Interaction*). Immediately after, the mother and her litter were removed from the home cage. One pup from the litter was randomly selected and returned to the home cage, and a 5-min recording was taken from this pup in isolation (R4: *Male/Female Pup Isolated)*. The pup was then reunited with his/her mother, and a littermate of the opposite sex was returned to the home cage for a 5-min recording (R5: *Female/Male Pup Isolated*). Individual pups were placed outside of the nest site during isolation recordings. The order of the sex of the pups recorded was counterbalanced within groups. The pups’ temperatures were measured during the isolation recording with an infrared temperature gun digital thermometer.

Day 2 of testing began with the mother injected intraperitoneally (IP) with 1.0 ml/kg of a solution that contained ketamine HCl (75.0 mg/mL), xylazine (7.5 mg/mL) and acepromazine maleate (1.5 mg/mL) before being placed into the testing room. Once the mother exhibited loss of palpebral/corneal and pedal withdrawal reflexes (~ 5 min), the litter was removed from the home cage and housed outside of the testing room. Fifteen minutes later, a 5-min recording of the anesthetized mother alone was taken to confirm the absence of maternal vocalizations. The litter was then returned and scattered in the home cage opposite to the nest, and a 15-min recording was taken (R6: *Litter with Mother Anesthetized*). During this recording, after 10 min had elapsed, the litter was grouped in the nest with the mother by a researcher, to compare litter vocalization levels before and after grouping.

### Experiment 2: Effect of the adenosine A_2A_ receptor antagonist MSX-3 on maternal behavior and affect of WKY mothers

This experiment examined the ability of the adenosine A_2A_ receptor antagonist MSX-3 to ameliorate the behavioral and vocal deficits of WKY mothers. Separate groups of SD and WKY postpartum female rats were randomly assigned to receive IP injections of either 1.0 mg/kg/ml of MSX-3 (Sigma Chemical, St. Louis, MO, USA) or same volume of corresponding vehicle. MSX-3 was freshly dissolved in 0.9% saline, which was also used as the vehicle condition. Twenty minutes before the maternal behavior test, both mother and litter were removed from the home cage, the mother received an injection of either MSX-3 or vehicle and was immediately returned to her home cage. The litter was housed in a small cage, lined with bedding and containing nest-building material from the maternal cage, outside of the testing room until testing. MSX-3 dose and injection time (1.0 mg/kg IP; 20 min before testing) were selected based on our previously published report showing that this dose effectively ameliorated haloperidol-induced maternal behavior deficits in SD mothers^66^.

### Maternal behavior test

Following 20 min of maternal separation, the entire litter was scattered in the home cage opposite to the nest, and the number, duration and latency of maternal behaviors were recorded continuously for 30 min, as previously described^63,66,108^. Other behaviors recorded included general exploration (line crosses and rearings), self-grooming and eating/drinking.

### Ultrasonic vocalization (USV) recording and analysis

USVs were recorded using a CM16/CMPA condenser ultrasound microphone connected via an Avisoft UltrasoundGate 116H acquisition device to a computer with Avisoft RECORDER software (sampling rate: 250 kHz; 16 bits; Avisoft Bioacoustics, Berlin, Germany). Acoustic analysis of the recorded .wav files was performed post hoc using Avisoft SASLab Pro software. Spectrograms were generated with a fast Fourier transformation (FFT) length of 512 points and a time window overlap of 75% (FlatTop window, 100% frame size). Correspondingly, spectrograms had a frequency resolution of 488 Hz and a temporal resolution of 0.512 ms. Analyses of USVs were performed blind with respect to experimental conditions by an experienced coder according to USV categories previously described^64^. A second coder independently analyzed a random subset of spectrograms and established inter-rater reliability higher than 90%. Acoustic features of each call, including duration, peak frequency and peak amplitude were measured by the automatic parameter measurement tool of the software.

### Statistics

A total of 41,622 USVs were manually detected and analyzed in this study. Based on visual inspection of the spectrograms, calls were categorized into 22 kHz or one of 10 50 kHz call categories^64^. In addition, USVs (< 1%) that did not fit any of the 12 categories were classified as “unclear”. Most frequency modulated (FM) categories, except for trills, had a low proportion of emission (less than 2%) by all groups and experiments and were combined into the FM category for statistical analysis. The following main call categories were used for final analysis: (1) negative 22 kHz call, and 50 kHz (2) flats, (3) shorts, (4) trills and (5) other FM calls. Acoustic data are expressed as mean ± standard error of the mean (SEM). Calling rates, proportional production, and acoustic parameters (frequency, duration) of these USV categories were analyzed using linear mixed models, with strain (SD and WKY), recording (R1–R6) and/or treatment (vehicle and MSX-3) as factors for relevant comparisons. Significant main effects and interactions were further analyzed using Tukey’s HSD tests. The χ^2^ goodness-of-fit test was used to analyze USV profiles from R1 and R2 recordings of mothers.

Behavioral data are expressed as mean ± SEM and were analyzed with independent-samples t test (Exp. 1) or two-way ANOVAs (Exp. 2) with strain (SD and WKY) and treatment (vehicle and MSX-3) as factors for relevant comparisons. Analyses of pups’ body weight and temperature were performed with two-way ANOVAs with strain (SD and WKY) and sex (female and male) as the between-subjects factors. Between-group categorical data comparisons were examined using χ^2^ test of independence and Fisher’s exact tests. Pearson’s tests were used for correlation analysis between number, duration or frequency of USVs and body weights, body temperature or maternal behavior. All analyses were performed using SPSS software (SPSS v25; IBM Corp., USA). Statistical significance was set at p < 0.05.

## Data Availability

The datasets generated and/or analyzed during the current study are available from the corresponding author on reasonable request.

## References

[1] Harwood K, McLean N, Durkin K (2007). First-time mothers' expectations of parenthood: What happens when optimistic expectations are not matched by later experiences?. Dev. Psychol..

[2] Murray L, De Pascalis L, Bozicevic L, Hawkins L, Sclafani V, Ferrari PF (2016). The functional architecture of mother–infant communication, and the development of infant social expressiveness in the first two months. Sci. Rep..

[3] Shoshani A, Yaari S (2022). Parental flow and positive emotions: Optimal experiences in parent–child interactions and parents’ well-being. J. Happiness Stud..

[4] Beardslee WR, Gladstone TRG, O’Connor EE (2011). Transmission and prevention of mood disorders among children of affectively ill parents: A review. J. Am. Acad. Child Adolesc. Psychiatry.

[5] Downey G, Coyne JC (1990). Children of depressed parents: An integrative review. Psychol. Bull..

[6] Field T, Healy BT, Goldstein S, Guthertz M (1990). Behavior-state matching and synchrony in mother–infant interactions of nondepressed versus depressed dyads. Dev. Psychol..

[7] Goodman SH (2007). Depression in mothers. Annu. Rev. Clin. Psychol..

[8] Goodman SH, Rouse MH, Connell AM, Broth MR, Hall CM, Heyward D (2011). Maternal depression and child psychopathology: A meta-analytic review. Clin. Child. Fam. Psychol. Rev..

[9] Lovejoy MC, Graczyk PA, O'Hare E, Neuman G (2000). Maternal depression and parenting behavior: A meta-analytic review. Meta-Analysis Clin. Psychol. Rev..

[10] O’Hara MW, McCabe JE (2013). Postpartum depression: Current status and future directions. Annu. Rev. Clin. Psychol..

[11] Braarud HC, Skotheim S, Høie K, Markhus MW, Kjellevold M, Graff IE, Berle JØ, Stormark KM (2017). Affective facial expression in sub-clinically depressed and non-depressed mothers during contingent and non-contingent face-to-face interactions with their infants. Infant Behav. Dev..

[12] Cicchetti D, Rogosch FA, Toth SL (1998). Maternal depressive disorder and contextual risk: Contributions to the development of attachment insecurity and behavior problems in toddlerhood. Dev. Psychopathol..

[13] Field T, Sandberg D, Garcia R, Vega-Lahr N, Goldstein S, Guy L (1985). Pregnancy problems, postpartum depression, and early mother–infant interactions. Dev. Psychol..

[14] Herrera E, Reissland N, Shepherd J (2004). Maternal touch and maternal child-directed speech: Effects of depressed mood in the postnatal period. J. Affect. Disord..

[15] Zlochower AJ, Cohn JF (1996). Vocal timing in face-to-face interaction of clinically depressed and nondepressed mothers and their 4-month-old infants. Infant Behav. Dev..

[16] Brudzynski, S. M. Handbook of ultrasonic vocalization. In *A Handbook into the Emotional Brain*, 1st ed (Levy, Niki, 2018).

[17] Panksepp J (2010). Affective neuroscience of the emotional BrainMind: Evolutionary perspectives and implications for understanding depression. Dialogues Clin. Neurosci..

[18] Brudzynski SM (2007). Ultrasonic calls of rats as indicator variables of negative or positive states: Acetylcholine-dopamine interaction and acoustic coding. Behav. Brain Res..

[19] Brudzynski SM (2013). Ethotransmission: Communication of emotional states through ultrasonic vocalization in rats. Curr. Opin. Neurobiol..

[20] Burgdorf JS, Brudzynski SM, Moskal JR (2020). Using rat ultrasonic vocalization to study the neurobiology of emotion: From basic science to the development of novel therapeutics for affective disorders. Curr. Opin. Neurobiol..

[21] Knutson B, Burgdorf J, Panksepp J (2002). Ultrasonic vocalizations as indices of affective states in rats. Psychol. Bull..

[22] Portfors CV (2007). Types and functions of ultrasonic vocalizations in laboratory rats and mice. J. Am. Assoc. Lab. Anim. Sci..

[23] Simola N, Granon S (2019). Ultrasonic vocalizations as a tool in studying emotional states in rodent models of social behavior and brain disease. Neuropharmacology.

[24] Wöhr M, Schwarting RK (2013). Affective communication in rodents: Ultrasonic vocalizations as a tool for research on emotion and motivation. Cell Tissue Res..

[25] Blanchard RJ, Blanchard DC, Agullana R, Weiss SM (1991). Twenty-two kHz alarm cries to presentation of a predator, by laboratory rats living in visible burrow systems. Physiol. Behav..

[26] Brudzynski SM (2011). Pharmacological and behavioral characteristics of 22kHz alarm calls in rats. Neurosci. Biobehav. Rev..

[27] Burgdorf J, Kroes RA, Moskal JR, Pfaus JG, Brudzynski SM, Panksepp J (2008). Ultrasonic vocalizations of rats (*Rattus norvegicus*) during mating, play, and aggression: Behavioral concomitants, relationship to reward, and self-administration of playback. J. Comp. Psychol..

[28] Burgdorf JS, Ghoreishi-Haack N, Cearley CN, Kroes RA, Moskal JR (2019). Rat ultrasonic vocalizations as a measure of the emotional component of chronic pain. NeuroReport.

[29] Dinh HK, Larkin A, Gatlin L, Piepmeier E (1999). Rat ultrasound model for measuring pain resulting from intramuscularly injected antimicrobials. PDA J. Pharm. Sci. Technol..

[30] Litvin Y, Blanchard DC, Blanchard RJ (2017). Rat 22 kHz ultrasonic vocalizations as alarm cries. Behav. Brain Res..

[31] Sales GD (1972). Ultrasound and aggressive behaviour in rats and other small mammals. Anim. Behav..

[32] Wöhr M, Borta A, Schwarting RKW (2005). Overt behavior and ultrasonic vocalization in a fear conditioning paradigm: A dose-response study in the rat. Neurobiol. Learn. Mem..

[33] Sánchez C (2003). R-citalopram attenuates anxiolytic effects of escitalopram in a rat ultrasonic vocalization model. Eur. J. Pharmacol..

[34] Thomas DA, Takahashi LK, Barfield RJ (1983). Analysis of ultrasonic vocalizations emitted by intruders during aggressive encounters among rats (*Rattus norvegicus*). J. Comp. Psychol..

[35] Barker DJ, Simmons SJ, Servilio LC, Bercovicz D, Ma S, Root DH, Pawlak AP, West MO (2014). Ultrasonic vocalizations: Evidence for an affective opponent process during cocaine self-administration. Psychopharmacology.

[36] Knutson B, Burgdorf J, Panksepp J (1998). Anticipation of play elicits high-frequency ultrasonic vocalizations in young rats. J. Comp. Psychol..

[37] Knutson B, Burgdorf J, Panksepp J (1999). High-frequency ultrasonic vocalizations index conditioned pharmacological reward in rats. Physiol. Behav..

[38] Panksepp J, Burgdorf J (2000). 50-kHz chirping (laughter?) in response to conditioned and unconditioned tickle-induced reward in rats: Effects of social housing and genetic variables. Behav. Brain Res..

[39] Simola N, Fenu S, Costa G, Pinna A, Plumitallo A, Morelli M (2012). Pharmacological characterization of 50-kHz ultrasonic vocalizations in rats: Comparison of the effects of different psychoactive drugs and relevance in drug-induced reward. Neuropharmacology.

[40] Willadsen M, Seffer D, Schwarting RK, Wöhr M (2014). Rodent ultrasonic communication: Male prosocial 50-kHz ultrasonic vocalizations elicit social approach behavior in female rats (*Rattus norvegicus*). J. Comp. Psychol..

[41] Wöhr M, Houx B, Schwarting RK, Spruijt B (2008). Effects of experience and context on 50-kHz vocalizations in rats. Physiol. Behav..

[42] Bell RW, Nitschke W, Bell NJ, Zachman TA (1974). Early experience, ultrasonic vocalizations, and maternal responsiveness in rats. Dev Psychobiol..

[43] Boulanger-Bertolus J, Rincón-Cortés M, Sullivan RM, Mouly A-M (2017). Understanding pup affective state through ethologically significant ultrasonic vocalization frequency. Sci. Rep..

[44] Brouette-Lahlou I, Vernet-Maury E, Vigouroux M (1992). Role of pups' ultrasonic calls in a particular maternal behavior in Wistar rat: Pups' anogenital licking. Behav. Brain Res..

[45] Brudzynski SM, Kehoe P, Callahan M (1999). Sonographic structure of isolation-induced ultrasonic calls of rat pups. Dev. Psychobiol..

[46] Brunelli SA, Shair HN, Hofer MA (1994). Hypothermic vocalizations of rat pups (*Rattus norvegicus*) elicit and direct maternal search behavior. J. Comp. Psychol..

[47] D'Amato FR, Scalera E, Sarli C, Moles A (2005). Pups call, mothers rush: Does maternal responsiveness affect the amount of ultrasonic vocalizations in mouse pups?. Behav. Genet..

[48] Ehret G (2005). Infant rodent ultrasounds—A gate to the understanding of sound communication. Behav. Genet..

[49] Farrell WJ, Alberts JR (2002). Stimulus control of maternal responsiveness to Norway rat (*Rattus norvegicus*) pup ultrasonic vocalizations. J. Comp. Psychol..

[50] Hashimoto H, Saito TR, Furudate S, Takahashi KW (2001). Prolactin levels and maternal behavior induced by ultrasonic vocalizations of the rat pup. Exp. Anim..

[51] Ihnat R, White NR, Barfield RJ (1995). Pup's broadband vocalizations and maternal behavior in the rat. Behav. Processes..

[52] Noirot E (1972). Ultrasounds and maternal behavior in small rodents. Dev. Psychobiol..

[53] Okabe S, Nagasawa M, Kihara T, Kato M, Harada T, Koshida N, Mogi K, Kikusui T (2013). Pup odor and ultrasonic vocalizations synergistically stimulate maternal attention in mice. Behav. Neurosci..

[54] Uematsu A, Kikusui T, Kihara T, Harada T, Kato M, Nakano K, Murakami O, Koshida N, Takeuchi Y, Mori Y (2007). Maternal approaches to pup ultrasonic vocalizations produced by a nanocrystalline silicon thermo-acoustic emitter. Brain Res..

[55] Bölükbas I, Mundorf A, Freund N (2020). Maternal separation in rats induces neurobiological and behavioral changes on the maternal side. Sci. Rep..

[56] Stevenson CW, Goodwin PE, Tunstall B, Spicer CH, Marsden CA, Mason R (2009). Neonatal maternal separation alters reward-related ultrasonic vocalizations in rat dams. Behav. Brain Res..

[57] Braw Y, Malkesman O, Merenlender A, Dagan M, Bercovich A, Lavi-Avnon Y, Weller A (2009). Divergent maternal behavioral patterns in two genetic animal models of depression. Physiol. Behav..

[58] Pardon MC, Gould GG, Garcia A, Phillips L, Cook MC, Miller SA, Mason PA, Morilak DA (2002). Stress reactivity of the brain noradrenergic system in three rat strains differing in their neuroendocrine and behavioral responses to stress: Implications for susceptibility to stress-related neuropsychiatric disorders. Neuroscience.

[59] Paré WP (1989). Stress ulcer susceptibility and depression in Wistar Kyoto (WKY) rats. Physiol. Behav..

[60] Tejani-Butt S, Kluczynski J, Paré WP (2003). Strain-dependent modification of behavior following antidepressant treatment. Prog. Neuropsychopharmacol. Biol. Psychiatry..

[61] López-Rubalcava C, Lucki I (2000). Strain differences in the behavioral effects of antidepressant drugs in the rat forced swimming test. Neuropsychopharmacology.

[62] Will CC, Aird F, Redei EE (2003). Selectively bred Wistar–Kyoto rats: An animal model of depression and hypercresponsiveness to antidepressants. Mol. Psychiatry..

[63] Winokur SB, Lopes KL, Moparthi Y, Pereira M (2019). Depression-related disturbances in rat maternal behaviour are associated with altered monoamine levels within mesocorticolimbic structures. J. Neuroendocrinol..

[64] Wright JM, Gourdon JC, Clarke PB (2010). Identification of multiple call categories within the rich repertoire of adult rat 50-kHz ultrasonic vocalizations: Effects of amphetamine and social context. Psychopharmacology.

[65] Shair HN, Masmela JR, Brunelli SA, Hofer MA (1997). Potentiation and inhibition of ultrasonic vocalization of rat pups: Regulation by social cues. Dev. Psychobiol..

[66] Pereira M, Farrar AM, Hockemeyer J, Müller CE, Salamone JD, Morrell JD (2011). Effect of the adenosine A2A receptor antagonist MSX-3 on motivational disruptions of maternal behavior induced by dopamine antagonism in the early postpartum rat. Psychopharmacology.

[67] Cloutier S, Wahl K, Baker C, Newberry RC (2014). The social buffering effect of playful handling on responses to repeated intraperitoneal injections in laboratory rats. J. Am. Assoc. Lab. Anim. Sci..

[68] Cierpial MA, Shasby DE, McCarty R (1987). Patterns of maternal behavior in the spontaneously hypertensive rat. Physiol. Behav..

[69] Myers MM, Brunelli SA, Squire JM, Shindeldecker RD, Hofer MA (1989). Maternal behavior of SHR rats and its relationship to offspring blood pressures. Dev. Psychobiol..

[70] Pereira, M., Khanna, V., Shiflett, M. & Morrell, J. I. Impairments in cognitive flexibility are associated with deficits in parenting in animal models of postpartum depression. In *Society for Neuroscience 44th Annual Meeting, Washington, DC, USA* (2014).

[71] Field T (2010). Postpartum depression effects on early interactions, parenting, and safety practices: A review. Infant Behav. Dev..

[72] Ierardi E, Ferro V, Trovato A, Tambelli R, Riva Crugnola C (2019). Maternal and paternal depression and anxiety: Their relationship with mother–infant interactions at 3 months. Arch. Womens Ment. Health..

[73] Ahrens AM, Ma ST, Maier EY, Duvauchelle CL, Schallert T (2009). Repeated intravenous amphetamine exposure: Rapid and persistent sensitization of 50-kHz ultrasonic trill calls in rats. Behav. Brain Res..

[74] Best LM, Zhao LL, Scardochio T, Clarke PB (2017). Effects of repeated morphine on ultrasonic vocalizations in adult rats: Increased 50-kHz call rate and altered subtype profile. Psychopharmacology.

[75] Haney M, Miczek KA (1994). Ultrasounds during agonistic interactions between female rats (*Rattus norvegicus*). J. Comp. Psychol..

[76] Miczek KA, Weerts EM, Vivian JA, Barros HM (1995). Aggression, anxiety and vocalizations in animals: GABAA and 5-HT anxiolytics. Psychopharmacology.

[77] Vivian JA, Miczek KA (1991). Ultrasounds during morphine withdrawal in rats. Psychopharmacology.

[78] Vivian JA, Miczek KA (1993). Diazepam and gepirone selectively attenuate either 20–32 or 32–64 kHz ultrasonic vocalization during aggressive encounters. Psychopharmacology.

[79] van Zyl PJ, Dimatelis JJ, Russell VA (2014). Changes in behavior and ultrasonic vocalizations during antidepressant treatment in the maternally separated Wistar–Kyoto rat model of depression. Metab. Brain Dis..

[80] Civic D, Holt VL (2000). Maternal depressive symptoms and child behavior problems in a nationally representative normal birthweight sample. Matern. Child Health J..

[81] Forsyth BW, Leventhal JM, McCarthy PL (1985). Mothers' perceptions of problems of feeding and crying behaviors. A prospective study. Am. J. Dis. Child..

[82] Luoma I, Koivisto AM, Tamminen T (2004). Fathers' and mothers' perceptions of their child and maternal depressive symptoms. Nord. J. Psychiatry..

[83] Murray L, Fiori-Cowley A, Hooper R, Cooper P (1996). The impact of postnatal depression and associated adversity on early mother–infant interactions and later infant outcome. Child Dev..

[84] Bettes BA (1988). Maternal depression and motherese: Temporal and intonational features. Child Dev..

[85] Lam-Cassettari C, Kohlhoff J (2020). Effect of maternal depression on infant-directed speech to prelinguistic infants: Implications for language development. PLoS ONE.

[86] Esser KH, Schmidt U (1989). Mother–infant communication in the lesser spear-nosed bat phyllostomus-discolor (Chiroptera, Phyllostomidae)—Evidence for acoustic learning. Ethology.

[87] Fernandez AA, Knörnschild M (2020). Pup directed vocalizations of adult females and males in a vocal learning bat. Front. Ecol. Evol..

[88] Golinkoff RM, Can DD, Soderstrom M, Hirsh-Pasek K (2015). (Baby)talk to me: The social context of infant-directed speech and its effects on early language acquisition. Curr. Dir. Psychol. Sci..

[89] Kitamura C, Burnham D (2003). Pitch and communicative intent in mother’s speech: Adjustments for age and sex in the first year. Infancy.

[90] Piazza EA, Iordan MC, Lew-Williams C (2017). Mothers consistently alter their unique vocal fingerprints when communicating with infants. Curr. Biol..

[91] Braw Y, Malkesman O, Merenlender A, Bercovich A, Dagan M, Overstreet DH, Weller A (2008). Withdrawal emotional-regulation in infant rats from genetic animal models of depression. Behav. Brain Res..

[92] Wöhr M, Schwarting RK (2008). Maternal care, isolation-induced infant ultrasonic calling, and their relations to adult anxiety-related behavior in the rat. Behav. Neurosci..

[93] Martins C, Gaffan EA (2000). Effects of early maternal depression on patterns of infant-mother attachment: A meta-analytic investigation. J. Child Psychol. Psychiatry..

[94] Skotheim S, Braarud HC, Hoie K, Markhus MW, Malde MK, Graff IE, Stormark KM (2013). Subclinical levels of maternal depression and infant sensitivity to social contingency. Infant. Behav. Dev..

[95] Oztop D, Uslu R (2007). Behavioral, interactional and developmental symptomatology in toddlers of depressed mothers: A preliminary clinical study within the DC:0–3 framework. Turk. J. Pediatr..

[96] Luecken LJ, Crnic KA, Gonzales NA, Winstone LK, Somers JA (2019). Mother–infant dyadic dysregulation and postpartum depressive symptoms in low-income Mexican-origin women. Biol. Psychol..

[97] Blumberg MS, Sokoloff G, Kent KJ (2000). A developmental analysis of clonidine’s effects on cardiac rate and ultrasound production in infant rats. Dev. Psychobiol..

[98] Branchi I, Santucci D, Alleva E (2001). Ultrasonic vocalisation emitted by infant rodents: A tool for assessment of neurobehavioural development. Behav. Brain Res..

[99] Elsner J, Suter D, Alder S (1990). Microanalysis of ultrasound vocalizations of young rats: Assessment of the behavioral teratogenicity of methylmercury. Neurotoxicol Teratol..

[100] Noirot E (1968). Ultrasounds in young rodents. II. Changes with age in albino rats. Anim. Behav..

[101] Okon EE (1972). Factors affecting ultrasound production in infant rodents. J. Zool..

[102] Geyer LA (1979). Olfactory and thermal influences on ultrasonic vocalization during development in rodents. Integr. Comp. Biol..

[103] Hofer MA, Shair H (1978). Ultrasonic vocalization during social interaction and isolation in 2-week-old rats. Dev. Psychobiol..

[104] Farrar AM, Pereira M, Velasco F, Hockemeyer J, Müller CE, Salamone JD (2007). Adenosine A(2A) receptor antagonism reverses the effects of dopamine receptor antagonism on instrumental output and effort-related choice in the rat: Implications for studies of psychomotor slowing. Psychopharmacology.

[105] Hauber W, Neuscheler P, Nagel J, Muller CE (2001). Catalepsy induced by a blockade of dopamine D1 or D2 receptors was reversed by a concomitant blockade of adenosine A2A receptors in the caudate putamen of rats. Eur. J. Neurosci..

[106] Worden LT, Shahriari M, Farrar AM, Sink KS, Hockemeyer J, Müller CE, Salamone JD (2009). The adenosine A2A antagonist MSX-3 reverses the effort-related effects of dopamine blockade: Differential interaction with D1 and D2 family antagonists. Psychopharmacology.

[107] Numan M, Numan MJ, Pliakou N, Stolzenberg DS, Mullins OJ, Murphy JM, Smith CD (2005). The effects of D1 or D2 dopamine receptor antagonism in the medial preoptic area, ventral pallidum, or nucleus accumbens on the maternal retrieval response and other aspects of maternal behavior in rats. Behav. Neurosci..

[108] Pereira M, Ferreira A (2006). Demanding pups improve maternal behavioral impairments in sensitized and haloperidol-treated lactating female rats. Behav. Brain Res..

[109] Silva MR, Bernardi MM, Felicio LF (2001). Effects of dopamine receptor antagonists on ongoing maternal behavior in rats. Pharmacol. Biochem. Behav..

[110] Silva MR, Bernardi MM, Cruz-Casallas PE, Felicio LF (2003). Pimozide injections into the nucleus accumbens disrupt maternal behaviour in lactating rats. Pharmacol. Toxicol..

[111] Stern JM, Keer SE (1999). Maternal motivation of lactating rats is disrupted by low dosages of haloperidol. Behav. Brain Res..

[112] Farrar AM, Segovia KN, Randall PA, Nunes EJ, Collins LE, Stopper CM, Port RG, Hockemeyer J, Müller CE, Correa M, Salamone JD (2010). Nucleus accumbens and effort-related functions: Behavioral and neural markers of the interactions between adenosine A2A and dopamine D2 receptors. Neuroscience.

[113] Fink JS, Weaver DR, Rivkees SA, Peterfreund RA, Pollack AE, Adler EM, Reppert SM (1992). Molecular cloning of the rat A2 adenosine receptor: Selective co-expression with D2 dopamine receptors in rat striatum. Brain Res. Mol. Brain Res..

[114] Ferre S, Fredholm BB, Morelli M, Popoli P, Fuxe K (1997). Adenosine-dopamine receptor-receptor interactions as an integrative mechanism in the basal ganglia. Trends Neurosci..

[115] Fuxe K, Agnati LF, Jacobsen K, Hillion J, Canals M, Torvinen M, Tinner-Staines B, Staines W, Rosin D, Terasmaa A, Popoli P, Leo G, Vergoni V, Lluis C, Ciruela F, Franco R, Ferre S (2003). Receptor heteromerization in adenosine A2A receptor signaling: Relevance for striatal function and Parkinson’s disease. Neurology.

[116] Hillion J, Canals M, Torvinen M, Casado V, Scott R, Terasmaa A, Hansson A, Watson S, Olah ME, Mallol J, Canela EI, Zoli M, Agnati LF, Ibanez CF, Lluis C, Franco R, Ferre S, Fuxe K (2002). Coaggregation, cointernalization, and codesensitization of adenosine A2A receptors and dopamine D2 receptors. J. Biol. Chem..

[117] Schiffmann SN, Jacobs O, Vanderhaeghen JJ (1991). Striatal restricted adenosine A2 receptor (RDC8) is expressed by enkephalin but not by substance P neurons: An in situ hybridization histochemistry study. J. Neurochem..

[118] Childs E, de Witt H (2008). Enhanced mood and psychomotor performance by a caffeine-containing energy capsule in fatigued individuals. Exp. Clin. Psychopharmacol..

[119] Simola N, Ma ST, Schallert T (2010). Influence of acute caffeine on 50-kHz ultrasonic vocalizations in male adult rats and relevance to caffeine-mediated psychopharmacological effects. Int. J. Neuropsychopharmacol..

[120] Simola N, Costa G, Morelli M (2016). Activation of adenosine A_2_A receptors suppresses the emission of pro-social and drug-stimulated 50-kHz ultrasonic vocalizations in rats: Possible relevance to reward and motivation. Psychopharmacology.

[121] Burgdorf J, Wood PL, Kroes RA, Moskal JR, Panksepp J (2007). Neurobiology of 50-kHz ultrasonic vocalizations in rats: Electrode mapping, lesion, and pharmacology studies. Behav. Brain Res..

[122] Demyttenaere K, De Fruyt J, Stahl SM (2005). The many faces of fatigue in major depressive disorder. Int. J. Neuropsychopharmacol..

[123] Nutt D, Demyttenaere K, Janka Z, Aarre T, Bourin M, Canonico PL, Carrasco JL, Stahl S (2007). The other face of depression, reduced positive affect: The role of catecholamines in causation and cure. J. Psychopharmacol..

[124] Rampello L, Nicoletti G, Raffaele R (1991). Dopaminergic hypothesis for retarded depression: A symptom profile for predicting therapeutical responses. Acta Psychiatr. Scand..

[125] Robinson RL, Stephenson JJ, Dennehy EB, Grabner M, Faries D, Palli SR, Swindle RW (2015). The importance of unresolved fatigue in depression: Costs and comorbidities. Psychosomatics.

[126] Stahl SM (2002). The psychopharmacology of energy and fatigue. J. Clin. Psychiatry..

[127] Figueira JSB, Pacheco LB, Lobo I, Volchan E, Pereira MG, de Oliveira L, David IA (2018). "Keep that in mind!" The role of positive affect in working memory for maintaining goal-relevant information. Front. Psychol..

[128] Isen AM, Reeve J (2005). The influence of positive affect on intrinsic and extrinsic motivation: Facilitating enjoyment of play, responsible work behavior, and self-control. Motiv. Emot..

[129] Nadler RT, Rabi R, Minda JP (2010). Better mood and better performance. Learning rule-described categories is enhanced by positive mood. Psychol. Sci..

